# A new fluorescent oxaliplatin(iv) complex with EGFR-inhibiting properties for the treatment of drug-resistant cancer cells†

**DOI:** 10.1039/d4qi03025g

**Published:** 2025-01-09

**Authors:** Monika Caban, Philipp Fronik, Alessio Terenzi, Anja Federa, Julia H. Bormio Nunes, Rastislav Pitek, Dominik Kirchhofer, Hemma H. Schueffl, Walter Berger, Bernhard K. Keppler, Christian R. Kowol, Petra Heffeter

**Affiliations:** a Center for Cancer Research and Comprehensive Cancer Center, Medical University of Vienna Borschkegasse 8a 1090 Vienna Austria petra.heffeter@meduniwien.ac.at +43 (0)1 40160-57557; b Institute of Inorganic Chemistry, Faculty of Chemistry, University of Vienna Waehringer strasse 42 1090 Vienna Austria christian.kowol@univie.ac.at +43-4277-52680 +43-1-4277-52609; c Department of Biological, Chemical and Pharmaceutical Sciences, University of Palermo Viale delle Scienze Ed. 17 90128 Palermo Italy; d Vienna Doctoral School in Chemistry, University of Vienna Waehringer Strasse 42 1090 Vienna Austria; e Research Cluster “Translational Cancer Therapy Research”, University of Vienna and Medical University of, Vienna Austria

## Abstract

Platinum chemotherapy is part of every second anticancer treatment regimen. However, its application is limited by severe side effects and drug resistance. The combination of platinum-based chemotherapeutics with EGFR inhibitors has shown remarkable synergism in clinical treatment. To enhance the tolerability of this combination, we designed a novel multi-action oxaliplatin-based platinum(iv) complex with an EGFR-inhibiting moiety (KP2749). KP2749 releases two independent cytotoxic agents upon reduction: oxaliplatin and the EGFR inhibitor KP2187, which was selected for its strong intrinsic fluorescence that became quenched upon complexation to metal ions. In particular, KP2749 demonstrated high stability and specific KP2187 release, with quenched fluorescent properties in its intact form, facilitating the investigation of its intracellular reduction. Notably, by exploiting its fluorescence, we demonstrated that intact KP2749 itself exhibited EGFR-inhibitory properties. Furthermore, subsequent experiments indicated that our complex was able to overcome resistance to oxaliplatin and EGFR inhibitors *in vitro* and in xenograft models *in vivo*. These effects were not only based on EGFR inhibition and DNA damage, but also improved cellular drug uptake. Finally, *in silico* docking analysis confirmed that the intact KP2749 complex had EGFR-binding properties, which were different from free KP2187. Consequently, these data suggested that the coordination of EGFR inhibitors to metal cores (like platinum) allow the fine-tuning of their EGFR-targeting properties. In conclusion, this study not only presents a new potential anticancer drug but also offers a novel fluorescent tool to study the intracellular drug release kinetics of platinum(iv) complexes.

## Abstract 2

KP2749 is a new anticancer agent that releases two independent cytotoxic agents upon reduction: oxaliplatin and the fluorescent EGFR inhibitor KP2187.

## Graphical abstract

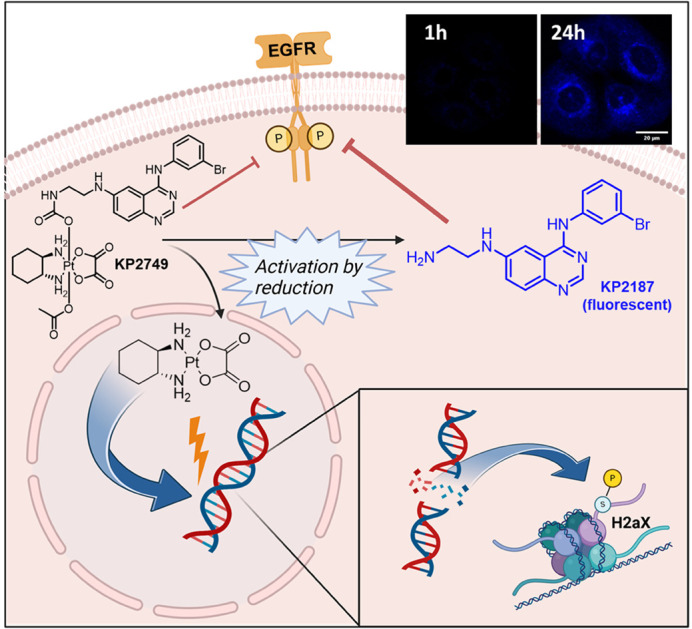

## Introduction

Nowadays, almost 50% of anticancer treatment regimens rely on systemic platinum-based chemotherapy.^1,2^ Non-small cell lung cancer (NSCLC) as well as head and neck, breast, ovarian, cervical, testicular, esophageal, colorectal and bladder cancers are some of the most prominent examples where systemic platinum-based chemotherapy finds application.^3–8^ Currently, the three platinum-based drugs approved worldwide for clinical use are cisplatin, carboplatin and oxaliplatin. Regarding their chemistry, all these drugs are in the +2 oxidation state and thus highly reactive. Their mode of action is mainly based on DNA damage (caused by DNA platination), which results in apoptotic cell death.^9,10^ In addition, oxaliplatin has been reported to expose cancer cells to the immune system by the induction of immunogenic cell death.^11^ Unfortunately, treatment with platinum drugs is limited by severe adverse effects, such as neurotoxicity, nephrotoxicity, ototoxicity, and gastrointestinal toxicity.^12–14^ Consequently, recent developments have shifted towards platinum(iv) complexes, which are usually considered prodrugs and thus less reactive in healthy tissues.^15^ Notably, these complexes have a different chemical structure with two additional axial positions in comparison to platinum(ii). This provides the possibility to attach further ligands which can not only help to fine-tune the reduction properties of the complex but also enable the introduction of targeting moieties or even bioactive drugs with new modes of action.

In addition to the adverse effects, the clinical use of platinum drugs is frequently hampered by intrinsic and acquired drug resistance.^16,17^ Here, besides reduced intracellular platinum levels (such as downregulation of the copper transporter CTR1) and elevated expression of glutathione, enhanced DNA repair is particularly important. One mechanism that leads to improved DNA repair capacities in cancer cells is the activation of the epidermal growth factor receptor (EGFR) signaling pathway.^18–20^ For instance, the combination of platinum drugs with EGFR tyrosine kinase inhibitors (EGFR-TKIs) has shown strong synergistic activities not only in preclinical models^21–24^ but also in clinical studies.^25–29^ Moreover, there are some platinum complexes in literature containing EGFR-TKIs (*e.g.*, erlotinib and gefitinib) as axial ligands.^30,31^

Clinically, small-molecular EGFR-TKIs (such as erlotinib, gefitinib or afatinib) are used mainly in lung cancer patients, where the EGFR signaling pathway is frequently constitutively activated by EGFR mutations (*e.g.*, deletion in exon 19 delE746-A750 or the point mutation L858R).^32^ However, despite initial response, the rapid occurrence of acquired EGFR-TKI resistance is observed, which is mediated by the EGFR point mutation T790M, activation of alternative signaling pathways such as c-MET, or uncoupling of the EGFR-downstream signaling pathway by the loss of tumor suppressors such as PTEN.^33^ This led, for example, to the development of third-generation EGFR inhibitors such as osimertinib, which specifically targets cells with T790M mutation.^34^ Noteworthy, the problem of EGFR-TKI resistance development remains and the search for efficient drug combination partners resulted in the recent approval of osimertinib together with carboplatin in EGFR-mutated NSCLC.^35–38^

**Fig. 1:**
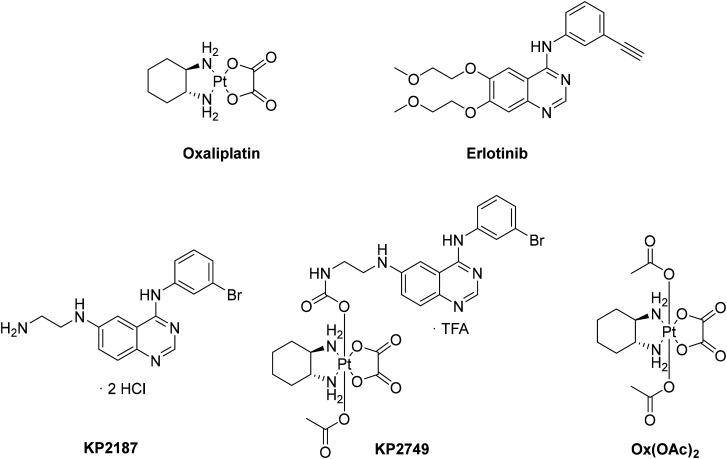
Chemical structures of the compounds tested in this study.

However, the severe toxicities of platinum-based therapy are a major drawback especially in combination settings. The limited usability in vulnerable patients is still to be overcome.^38^ In the study presented here, we designed a novel drug in which KP2187, an EGFR inhibitor ligand with remarkable *in vitro* and *in vivo* efficacy previously developed by our group (Fig. 1),^39^ was attached to an oxaliplatin-based platinum(iv) core (KP2749). This EGFR inhibitor was specifically selected due to its strong intrinsic fluorescent properties, which have been previously demonstrated to be quenched upon complexation with metal ions such as cobalt.^40^ Indeed, the platinum(iv) complex KP2749 not only exhibited high stability and specific KP2187 release upon reduction, but also showed quenched fluorescence compared to the free ligand. Subsequently, we could show that the intact complex is a potent EGFR inhibitor itself, which efficiently transports the platinum inside the cancer cells. This results in promising anticancer activity *in vitro* and *in vivo*, also against diverse drug-resistant cancer cell models.

## Results and discussion

### Synthesis, aqueous stability and reduction properties

Recently, D. Gibson and his co-workers have published an elegant synthetic method to couple amines to a platinum core.^41,42^ With the use of *N*,*N*′-disuccinimidyl carbonate, the axial hydroxido group at the platinum core can be activated and amine-containing ligands with a variety of structures and scaffolds can easily be attached *via* the formation of a “carbamato” moiety. We applied this method using the free amino group of KP2187 (synthesized in 5 steps according to Karnthaler-Benbakka *et al.*^40^) and the oxaliplatin precursor OxOH/OAc to obtain KP2749, an EGFR inhibitor-releasing platinum(iv) prodrug (Fig. 1). Oxaliplatin, as well as erlotinib and KP2187, were used as controls, together with Ox(OAc)_2_, a platinum(iv) complex with innocent acetato-ligands (Fig. 1).

**Fig. 2:**
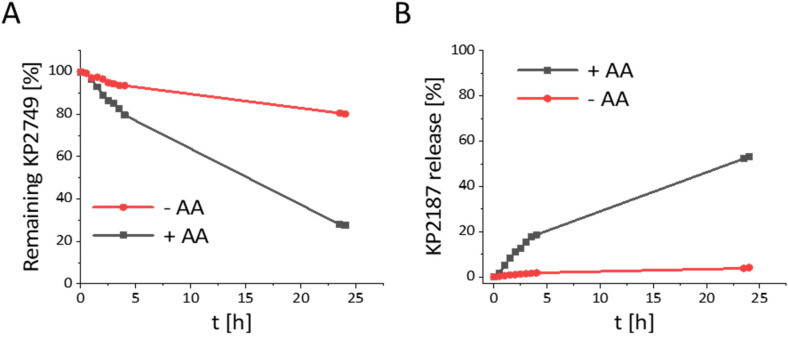
(A) Stability and (B) ligand release from KP2749 (500 μM) in PB (10% dimethylformamide) measured by UHPLC, with or without a 10-fold excess of ascorbic acid (AA).

The aqueous stability of KP2749 was assessed by ultra-high performance liquid chromatography (UHPLC) after incubation in phosphate buffer (PB) at pH 7.4. Interestingly, we observed a 20% decrease in the peak intensity after 24 h at 20 °C (Fig. 2A). However, only <5% release of KP2187 was detected (Fig. 2B). Further analysis by HPLC-MS revealed that this decrease in the KP2749 peak can be attributed to the ring-opening hydrolysis of the oxalato-ligand, yielding the mono-hydroxido platinum(iv) species (ESI Fig. S1†). This phenomenon has been already reported by our group for model platinum(iv) species at 37 °C.^43^ However, it was so far not observed at only 20 °C. Under reductive conditions with a 10-fold excess of ascorbic acid, a release of ∼50% of KP2187 could be observed after 24 h (Fig. 2B). This substantial effect was unexpected, as similar oxaliplatin-based prodrugs are usually reduced by <30% at this time point under similar conditions.^44,45^ Our observation can most probably be explained by the accelerated reduction rates of hydrolyzed oxaliplatin(iv) cores in comparison to the intact native complex.^43^

### Activity of the new drugs in cells with different EGFR mutation status and acquired EGFR-TKI drug resistance

To evaluate KP2749 for its anticancer activity, a panel of different cell models with either oxaliplatin or erlotinib sensitivity/resistance was tested. Thus, cell lines from 4 different categories were used (Table 1 and ESI Fig. S2, S3†): (1) different EGFR/wt expression status (A431 and RU-MH cells with erlotinib sensitivity due to EGFR/wt overexpression, MCF-7 cells with low EGFR/wt, which are erlotinib-unresponsive), (2) with high sensitivity to EGFR inhibition due to EGFR-TKI-sensitizing mutation (PC9 and HCC827), (3) resistance to EGFR inhibition (H1650 with intrinsic EGFR-TKI resistance due to PTEN loss,^46,47^ H1975 with EGFR-TKI resistance due to secondary EGFR mutation T790M^32^ as well as two HCC827 subclones with acquired resistance to gefinitib or erlotinib, based on c-MET overexpression),^39^ and (4) the EGFR-TKI-resistant colon cancer model HCT116 (KRAS mutation) together with its oxaliplatin-resistant subclone HCT116/OxR.

**Table 1:** Anticancer activity of the drugs against different cancer cell lines after 72 h. Statistical significance calculations are shown in ESI Fig. S4†

Cell line	EGFR status	KP2749	KP2187	Erlotinib	Oxaliplatin
		Mean ± SD (μM)			
A431	EGFR/wt overexpression	5.2 ± 1.3	6.8 ± 0.1	12.8 ± 3.8	9.50 ± 0.7
RU-MH	EGFR/wt overexpression	4.6 ± 0.3	8.5 ± 1.9	23.0 ± 2.6	4.7 ± 0.2
MCF-7	Low EGFR/wt	11.1 ± 1.7	9.1 ± 2.5	>25	9.9 ± 2.1
PC-9	EGFR overexpression; sensitizing mutation (delE746-A750)	0.7 ± 0.1	0.5 ± 0.02	0.7 ± 0.2	4.4 ± 1.5
HCC827	EGFR overexpression; sensitizing mutation (delE746-A750)	0.15 ± 0.07	0.04 ± 0.00	0.17 ± 0.02	0.58 ± 0.22
HCC827/erlo	EGFR overexpression; sensitizing mutation (delE746-A750), MET amplification	>10	>10	>10	0.6 ± 0.4
HCC827/gefi	EGFR overexpression; sensitizing mutation (delE746-A750), MET amplification	5.9 ± 2.1	>10	>10	4.9 ± 2.7
H1650	EGFR overexpression; sensitizing mutation (delE746-A750); PTEN loss	5.5 ± 0.8	10.0 ± 0.6	>25	6.1 ± 1.9
H1975	Sensitizing mutation (L858R) resistance mutation (T790M)	2.5 ± 0.9	12.9 ± 0.7	24.7 ± 0.4	2.7 ± 0.4
HCT116	Low EGFR/wt expression; Kras mutation	9.6 ± 0.0	8.2 ± 0.1	>25	2.9 ± 1.9
HCT116/OxR	EGFR/wt; Kras mutation, resistant to oxaliplatin	10.4 ± 2.0	10.5 ± 2.7	n.d.	>20

With regard to the reference drugs, cells with known dependence on the EGFR-signaling pathway (PC-9 and HCC827) responded with high sensitivity (IC_50_ in the nM range) to both EGFR inhibitors (erlotinib and KP2187). As expected, all cell models with low EGFR expression or changes associated with resistance to TKI treatment were resistant to erlotinib up to the highest tested concentration (25 μM, limit of solubility). Also, KP2187 was less effective in these cell models. However, with the exception of HCC827 subclones, KP2187 was always superior to erlotinib with IC_50_ values in most cases about 2-fold lower than the approved EGFR inhibitor. With regard to oxaliplatin, IC_50_ values were observed in general in the low μM range. Only HCC827 cells were also highly sensitive to oxaliplatin (IC_50_ of 0.6 μM), while strong drug resistance (>7-fold) was confirmed in the HCT116/OxR cells (ESI Fig. S4†). Notably, the dual-action drug (KP2749), although having IC_50_ values in the low μM range, differed in its activity profile from all three reference compounds. In more detail, the resistance to one of the free parental compounds did not result in cross-resistance to KP2749, especially in case of the resistant cell models H1650, H1975 and HCT116/OxR. Only the c-MET-overexpressing HCC827 subclones were found to be unresponsive to KP2749 after 72 h treatment. The data are in good agreement with a recent literature report by Li *et al.* on a platinum(iv) complex releasing an EGFR inhibitor, which was also able to break cisplatin resistance in A549/CDDP cells.^31^

**Fig. 3:**
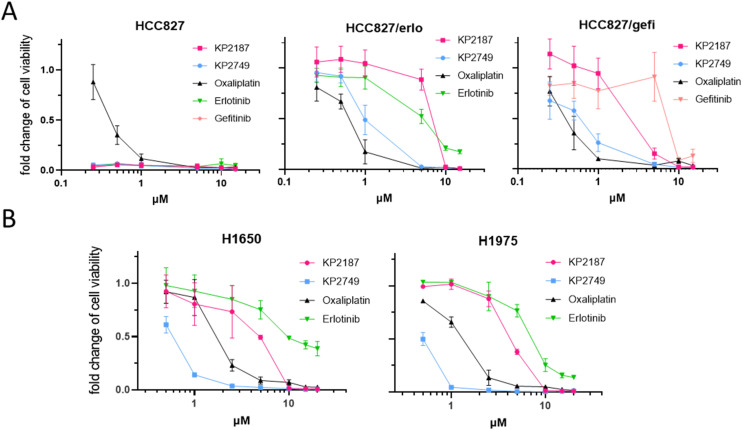
Long-term cytotoxicity in EGFR-TKI-resistant cell models. (A) Clonogenic survival of HCC827, its corresponding resistant sublines, and (B) the resistant cell models H1650 and H1975 after 10 days of incubation. The long-term exposure to the tested compounds was followed by crystal violet staining. Crystal violet-associated fluorescence was measured with a Typhoon Scanner and the signal intensity quantified with ImageJ. All drug response curves were normalized to untreated control cells. The figure shows a representative graph of three independently performed experiments delivering similar results. The given values are shown as means ± SD.

To gain more insights into the long-term effects of KP2749, especially against the TKI-resistant cell models, clonogenic assays after 10 days were performed in all HCC827 clones as well as in H1650 and H1975 cells. As shown in Fig. 3 and Table 2, upon long-term exposure, KP2749 was able to break even the strong drug resistance of the c-MET-overexpressing sublines HCC827/erlo and HCC827/gefi, displaying effectivity in the range of free oxaliplatin. Noteworthy, in the case of H1650 and H1975, KP2749 was not only better than erlotinib, gefitinib and KP2187 but also exerted superior activity to the free platinum(ii) complex oxaliplatin.

**Table 2:** IC_50_ values of long-time treated cells after 10 days of drug incubation

Cell line	KP2749	KP2187	Oxaliplatin	Erlotinib	Gefitinib
Mean ± SD (μM)					
HCC827	0.13 ± 0.003	0.13 ± 0.003	0.45 ± 0.074	0.13 ± 0.004	0.13 ± 0.001
HCC827/erlo	1.34 ± 0.53	7.22 ± 0.22	0.68 ± 0.02	7.59 ± 3.13	n.t.
HCC827/gefi	0.69 ± 0.17	2.64 ± 0.86	0.49 ± 0.16	n.d.	9.2 ± 1.67
H1650	0.60 ± 0.27	3.10 ± 1.53	1.91 ± 0.73	10.28 ± 1.35	n.d.
H1975	0.55 ± 0.11	4.31 ± 1.53	1.21 ± 0.31	6.51 ± 1.04	n.d.

**Fig. 4:**
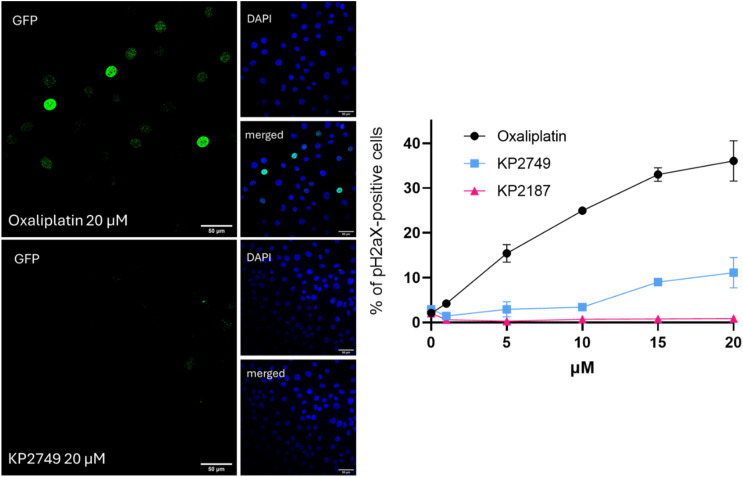
KP2749-related DNA damage after 24 h. H1650 cells were seeded into 96-well imaging black plates and treated with the indicated compounds for 24 h. The cells were PFA-fixed and immunofluorescently stained for γH2aX. Cell nuclei were counterstained with DAPI. An image-based cytometry method was used to analyze the γH2aX (foci)-positive cell populations *via* Evident ScanR software. For each well, 9 regions of interest were analyzed. The signal of γH2aX was quantified and calculated for individual wells. The shown values are percentages of γH2aX-positive cells ± SD.

To shed light on the mode of action of KP2749, therapy-induced DNA damage was analyzed by an image-based cytometry method using γH2aX as a marker (Fig. 4). Unexpectedly, in contrast to the viability and the clonogenicity data above, KP2749 treatment for 24 h induced only minor DNA damage compared to oxaliplatin, suggesting that DNA platination could explain only part of the effects observed in the cell culture experiments on the EGFR-TKI-resistant cell lines.

### Lipophilicity and cellular drug accumulation

**Fig. 5:**
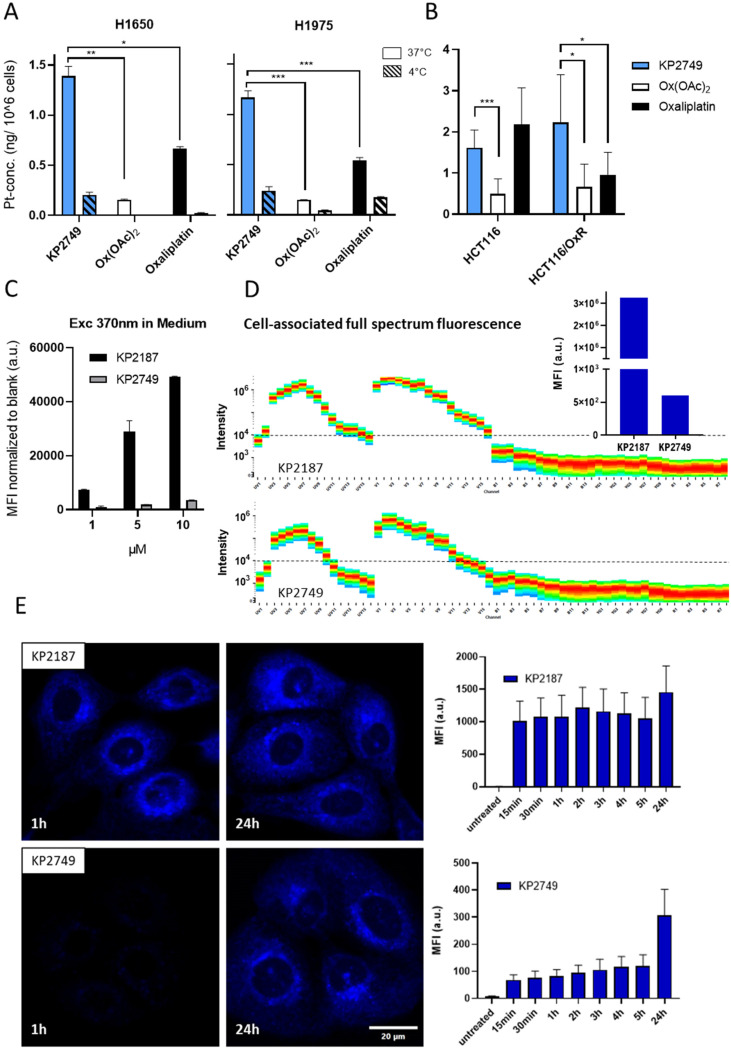
Drug uptake and ligand release *in vitro*. (A and B) The indicated cells were treated with 10 μM of the indicated drugs for 5 h. Cells were lysed with 67% HNO_3_. The platinum concentration was measured *via* ICP-MS and the gained values were blank corrected and normalized to the cell number. Data shown are means ± SD of three pooled independent experiments performed in triplicates. Statistical significance was calculated by two-way ANOVA and Tukey's multiple comparison test. (C) Intrinsic fluorescence properties of KP2187 and KP2749 measured under cell-free conditions in cell culture medium using a Tecan plate reader. (D) Cell-associated fluorescence in H1650 cells after 1 h of incubation with 5 μM of KP2187 or KP2749 were analyzed using the full spectrum flow cytometry system Cytek Aurora. The inset shows the mean fluorescence intensities (MFI) of both drugs quantified from the full fluorescence spectrum (range 365 nm–829 nm). (E) Live-cell imaging experiment on H1650 cells. Cells were treated with 5 μM for the indicated time points. Then, high-throughput imaging was performed on a spinning disk confocal microscope, and the collected data was used for neuronal network training, followed by quantification of the fluorescence intensity using the ScanR software.

To better understand the results obtained in the cell culture experiments, cellular uptake and intracellular distribution of KP2749 were investigated. As a first step, the lipophilicity of the drugs was analyzed using the shake-flask method, followed by the determination of platinum concentrations by ICP-MS. The platinum(iv) complex Ox(OAc)_2_ (which has no EGFR inhibitor attached) as well as oxaliplatin were used as references. The measurements revealed that KP2749 with a log *D*_7.4_ at +1.03 was significantly more lipophilic than oxaliplatin (−1.63) and Ox(OAc)_2_ (−1.86^48^). However, the lipophilicity was lower than that of KP2187 (+1.86^49^). These results suggested that KP2749 could have an improved drug uptake profile compared to Ox(OAc)_2_ and oxaliplatin. Thus, the cellular uptake was evaluated in H1650 and H1975 cells, as well as the oxaliplatin-resistant HCT116 model (HCT116/wt and HCT116/OxR), by ICP-MS after 5 h of treatment. In good agreement with previous data, Ox(OAc)_2_ uptake was about 3-4-fold lower compared to oxaliplatin (Fig. 5A and B), showing that oxaliplatin-releasing platinum(iv) prodrugs are frequently characterized by a reduced intracellular accumulation.^50,51^ In contrast, KP2749 uptake was not only superior to the one of Ox(OAc)_2_ but in most cell lines even to the oxaliplatin uptake. This is particularly notable in the HCT116 model, where (in contrast to oxaliplatin) no impact of the drug resistance on drug uptake was detected. Interestingly, a similar superior resistance-independent drug uptake was also reported for the platinum(iv)-EGFR inhibitor complex from Li *et al.*^31^

To evaluate, whether these effects were solely based on the enhanced lipophilicity of the drug(s), the experiments in H1650 and H1975 were also performed at 4 °C, which stops all active molecular transport processes (Fig. 5A). The collected data showed a distinctly reduced drug uptake at reduced temperature, indicating that KP2749 is probably taken up *via* an active transport mechanism. Overall, this shows that the attachment of KP2187 to the platinum core distinctly alters its cellular uptake profile.

Since ICP-MS only allows the assessment of total platinum content without information on the uptake of the attached ligands, we exploited the specific intrinsic blue-fluorescent properties of KP2187, which were discovered during our previous studies.^40^ Noteworthy, the fluorescence was quenched by coordination to cobalt,^40^ and we speculated that similar effects could occur with complexation to platinum. Indeed, already when measuring the fluorescence intensities under cell-free conditions, the quenched fluorescence was observed for KP2749 in comparison to KP2187 (Fig. 5C). This was also confirmed on a cellular level in H1650 cells using the full spectrum flow cytometry system Cytek Aurora (Fig. 5D). In detail, KP2749 had a >5000-fold lower mean fluorescence intensity (MFI) than KP2187 (insert in Fig. 5D). As a next step, we exploited these fluorescent traits to follow the intracellular behavior of KP2749 over time. To this end, live-cell experiments with spinning disk confocal microscopy (Fig. 5E) were performed, which revealed that despite the strong cellular uptake of KP2749 (indicated by the ICP-MS measurements), only minor KP2187-associated fluorescence was detected for the first 5 h. This indicated that KP2749 rapidly enters the cell in the form of an intact complex. Upon prolonged incubation time (up to 24 h), an increase in cellular fluorescence could be observed in KP2749-treated cells, which enabled us to visualize the slow continuous ligand release inside the cancer cells.

### EGFR-inhibitory potential of KP2749 in cell culture

**Fig. 6:**
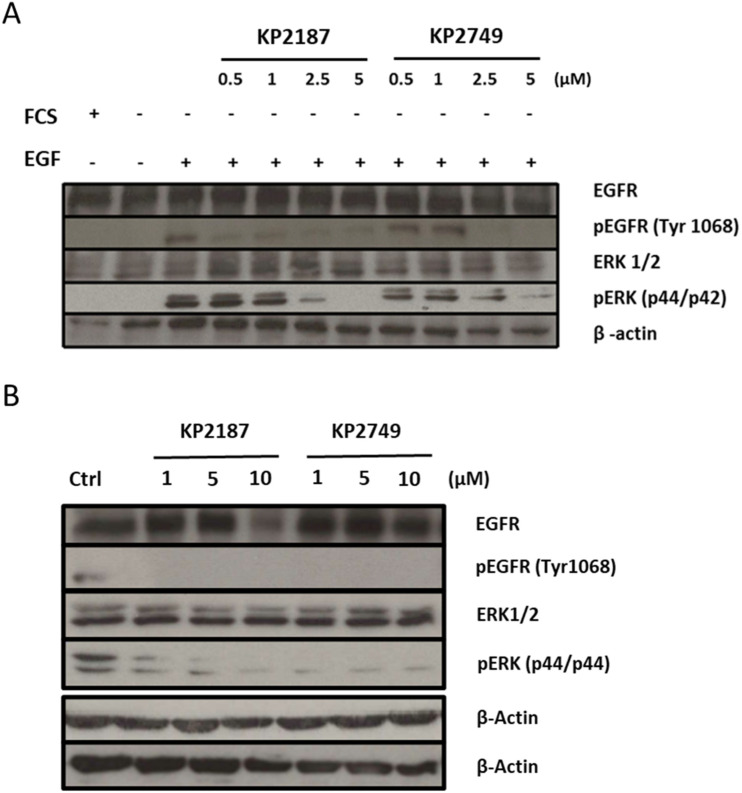
*In vitro* inhibition of EGFR signaling in TKI-sensitive A431 and TKI-resistant H1650 cell model. Western blot analysis of total protein extracts of tested cell models was performed (20 μg of protein sample loaded). (A) A431 cells were cultured with (+) or without (−) 10% FCS and treated for 4 h with indicated concentrations of KP2187 and KP2749. The EGFR expression was stimulated for 10 min with 50 ng mL^−1^ EGF (+). (B) H1650 treated for 24 h with 1, 5 or 10 μM of the indicated drugs. Starvation and EGF stimulation were not necessary in this model due to its EGFR mutation. β-Actin served as the loading control.

In order to evaluate the EGFR inhibition potential of KP2749 in comparison to free KP2187, Western blot analysis of the EGFR-signaling pathway (phosphorylation of EGFR at position Tyr1068 and down-stream ERK) were performed in A431 and H1650 cells. In case of A431, which was characterized by the overexpression of wild-type EGFR, 24 h serum starvation and activation of EGFR signaling by EGF stimulation were necessary to enhance the signal of EGFR phosphorylation for Western blot detection. In contrast, H1650 cells harbor a mutation of the EGFR, leading to constitutively active signaling. Consequently, no EGF stimulation was needed in this case. As shown in Fig. 6A and B, KP2749 was able to inhibit the phosphorylation of EGFR (Tyr 1068) as well as of the downstream- signaling molecule ERK1/2 (Thr202/Tyr204) in both tested cell models. Considering the experiment in A431 cells (with only a short incubation time of 4 h), these results were unexpected, since the chemical characterization, the distinct differences in the drug uptake, and intracellular ligand release experiments (shown above) together indicated high stability of the complex under cell culture conditions for several hours. Consequently, we hypothesized that the intact platinum(iv) complex could have EGFR-inhibiting properties itself although less efficient than free KP2187.

### Characterization of the intact KP2749 complex as EGFR inhibitor by molecular docking studies

**Fig. 7:**
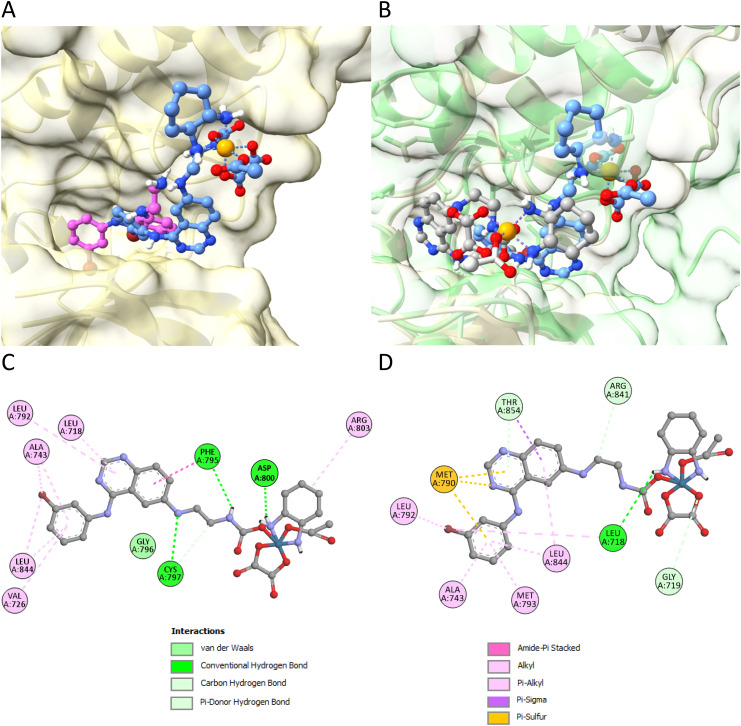
*In silico* docking studies of the compounds to the EGFR molecule. (A) 3D representation of EGFR/wt (PDBid: 4G5J) interacting with KP2187 (in pink) or KP2749 (in blue). (B) Comparison of the 3D binding of KP2749 (in blue) to EGFR/wt (PDBid: 4G5J, gold surface) with KP2749 (in grey) bound to the mutant EGFR (PDBid: 5CAS, green surface). Results were obtained by molecular docking performed using AutoDock. (C) and (D) Schematic built with Biovia Discovery Studio Visualizer of protein–ligand interactions for wild-type (C) and mutant (D) EGFR in complex with KP2749. No spatial information can be inferred from the 2D diagrams. Interaction types are depicted in the legend.

To evaluate whether the intact KP2749 is able to interact with the EGFR, *in silico* docking studies were performed. Recently, we used molecular docking to demonstrate that KP2187 effectively fits into the ATP-binding pocket of the human wild-type EGFR.^39^ Notably, KP2187 can take two distinct orientations, with one aligning closely with the orientation of the EGFR inhibitor afatinib, the original ligand, which was co-crystallized with the target (PDBid: 4G5J). In comparison to KP2187 (Fig. 7A; pink structure), the optimal pose taken by KP2749 (Fig. 7A; blue structure) reveals the quinazoline moiety positioned in the outer region of the ATP-binding pocket, potentially due to steric hindrance. However, KP2749 is situated within the protein cleft, sharing interactions with the same amino acid residues as KP2187, including Leu718, Ale743, Leu844, Val726, Gly796, and Asp800 (Fig. 7C and Fig. S6†). The binding energy values (Table 3) indicate that KP2749 exhibits a stronger binding affinity to wild-type EGFR compared to KP2187. This enhanced affinity is likely attributed to additional electrostatic interactions involving other residues, such as the cyclohexane moiety of oxaliplatin acting as an anchoring point with the Arg803 residue. Notably, Montagner and collaborators recently demonstrated a similar effect with Pt(iv) prodrugs derived from cisplatin and functionalized with derivatives of the tyrosine kinase inhibitors imatinib and nilotinib. These prodrugs were shown to effectively interact with and occupy the pocket of the PDGFR-α receptor, highlighting their potential as targeted therapeutic agents.^52^

Docking free energy values for the binding of the indicated molecules

**Table:** 

PDBid: 4G5J (WT EGFR)	Kcal mol^−1^
KP2187	−8.13^a^ (−6.99)
KP2749	−9.71

**Table:** 

PDBid: 5CAS (mutated EGFR)	Kcal mol^−1^
KP2187	−7.73^a^ (−7.52)
KP2749	−8.89

a No overlap with afatinib original pose in 4G5J.

In addition, we conducted docking calculations for both KP2187 and KP2749 using the mutant EGFR kinase domain T790M-L858R (TMLR) (PDBid: 5CAS, green surface),^53^ which is known for its resistance-conferring properties against many first and second generation EGFR-TKIs.^54–56^ To this end, the structure of wt-EGFR (PDBid: 4G5J, golden surface) was aligned with the one of the mutant EGFR (PDBid: 5CAS, green surface). Then, the two drugs were docked into the mutant EGFR (Fig. 7B). The findings reveal that both the ligand and the metal complex maintain interactions with the ATP-binding site of the protein. While the orientation of KP2187 remains relatively unchanged, keeping the quinazoline fragment in a consistent position regardless of whether bound to wild-type or mutant EGFR (Fig. S6†), KP2749 appears to better fit into the mutated protein cleft as the platinum(iv) center is positioned deeper inside the pocket (blue *vs.* gray structure in Fig. 7B). In comparison to the wild-type protein, when bound to the mutant EGFR, KP2749 displayed non-covalent interactions, involving its π-system with the sulfur atom of the Met790 residue (Fig. 7D). Intriguingly, KP2749 bound to the mutant EGFR exhibits greater stability than KP2187 (Table 3).

### EGFR inhibition potential, drug distribution and anticancer activity *in vivo*

**Fig. 8:**
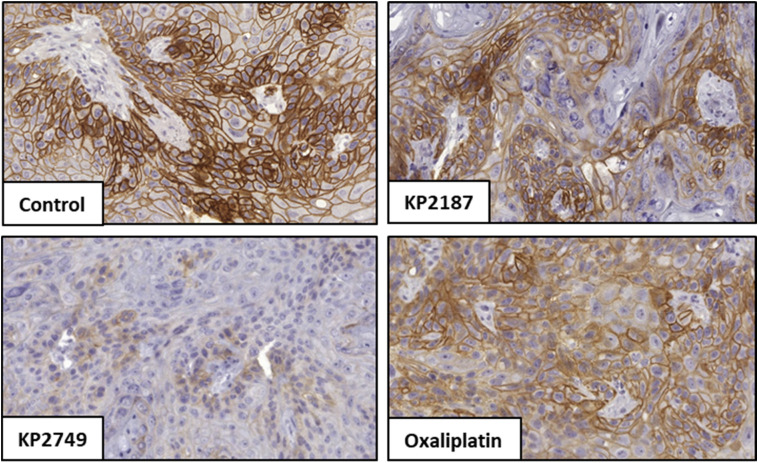
Inhibition of EGFR/wt phosphorylation in A431 xenografts *in vivo*. A431 cells were grown s.c. in C.B.-17/Scid mice. When the tumors reached a size of ∼300–500 mm^3^, the animals were treated intraperitoneally (i.p.) with a single equimolar dose of either KP2187 (9.8 mg kg^−1^), KP2749 (23.5 mg kg^−1^), or oxaliplatin (9 mg kg^−1^, MTD). After 24 h, the tumors were collected, formalin-fixed and paraffin-embedded. Finally, 3.5 μm-thin slices were prepared and immunohistochemically stained for p-EGFR (Tyr1068) and nuclei using a DAB detection system and hematoxylin, respectively. Representative images of stained tumor sections of untreated (control) and treated mice.

To investigate if the inhibition of EGFR by KP2749 translates into an *in vivo* situation, A431-bearing SCID mice were treated once with the compounds in a preliminary experiment, and tumor sections were immunohistochemically stained for p-EGFR (Tyr1068) (Fig. 8). Indeed, the stains indicated that KP2749 was able to inhibit EGFR/wt phosphorylation by an even higher extent than KP2187.

**Fig. 9:**
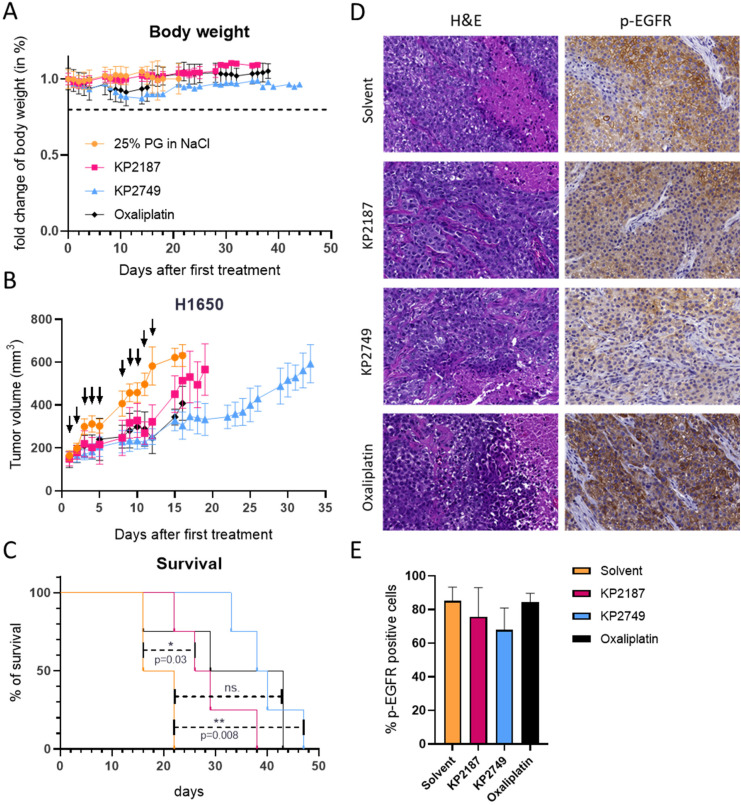
Tolerability and antitumor activity against H1650 xenografts *in vivo*. H1650 cells were grown s.c. in male C.B.-17/SCID mice. When the tumors reached the size of ∼175 mm^3^, animals were treated i.p. either twice per week with oxaliplatin (9 mg kg^−1^) or three times per week with KP2749 (49 mg kg^−1^) and KP2187 (20 mg kg^−1^) for two weeks. (A) The body weight was monitored throughout the whole experiment and measured on 5 consecutive days until mice were sacrificed. (B) Impact on tumor growth; data are presented as mean ± SEM. Curves are always shown until the first of the mice in the respective test group had to be sacrificed. (C) The overall survival of the animals is depicted *via* a Kaplan–Meier curve. Statistical significance was tested by log-rank test and Mantel–Cox post-test. (D) In a second experiment with the same treatment setting, the tumors were collected on the last day of therapy after treatment and sections were prepared for histological analysis (H&E stain) and immunohistochemically stained against p-EGFR with hematoxylin as the counterstain. Representative images of the H&E (general morphology) and p-EGFR stains (Tyr1068) are shown. (E) Quantification of the p-EGFR stains by HALO software.

Encouraged by these data, we performed a therapy experiment in H1650 xenografts. To this end, the mice were treated for two weeks, either twice per week with oxaliplatin at the maximum tolerated dose (MTD) of 9 mg kg^−1^ or three times per week with KP2749 or KP2187. In this experiment, due to the very good tolerability, KP2749 and KP2187 were applied at a higher (equimolar) dose of 49 mg kg^−1^ and 20 mg kg^−1^, respectively. The acceptable toxicity profile of all therapy schemes with only some transient loss in the body weight in the platinum-treated groups can also be seen in Fig. 9A. The good tolerability of KP2749 is noteworthy, as the total administered platinum dose was ∼3-fold higher than the one of the oxaliplatin scheme (given at MTD). With regard to the treatment efficiency, all compounds reduced tumor progression (Fig. 9B) and thus enhanced the overall survival of the animals (Fig. 9C). In good agreement with our *in vitro* data, KP2749 was superior to both KP2187 and oxaliplatin. In a second experiment, the tumors were collected on the last day of treatment and stained for the EGFR phosphorylation levels. In line with the enhanced anticancer activity of KP2749 (and the results observed in the A431 xenografts), also a stronger inhibition of EGFR phosphorylation than in KP2187-treated animals was observed (Fig. 9D and E).

Finally, we wanted to investigate whether the attachment of KP2187 to the platinum core also improves the anticancer activity against cells harboring the T790M mutation, as indicated by our docking studies. Consequently, H1975-bearing C.B.-17/SCID mice were treated with our drug panel and the impact on tumor growth was followed by caliper measurements (ESI Fig. S7†). The tumor model was widely unresponsive to KP2187 and oxaliplatin, as indicated by the non-significant impact on tumor growth and overall survival in comparison to the solvent-treated animals. In contrast, KP2749 significantly reduced tumor growth and prolonged the mean overall survival of the H1975-treated mice from 29 to 42 days (ESI Fig. S7A and B†). Although this effect is doubtlessly weaker than the response to osimertinib treatment,^57^ it demonstrates that the attachment to platinum could indeed be an interesting new strategy to modulate and fine-tune the EGFR-binding spectrum of TKIs, which should be considered in future studies.

## Conclusion

Drug resistance and adverse effects are serious issues in the treatment of cancer since intrinsic or acquired resistance often requires modification or even termination of therapeutic approaches including treatment with platinum(ii) complexes and EGFR inhibitors. Thus, it is of great interest to develop new drugs that can circumvent these resistance mechanisms, while at the same time showing reduced side effects. Considering recent studies that have shown clinical synergism between EGFR inhibitors and platinum-based chemotherapies (with the recent approval of the combination of osimertinib with carboplatin for advanced lung cancer),^25–29,58^ the coupling of an EGFR inhibitor to a platinum core in one molecule might offer a promising strategy to further enhance the tumor specificity and thus the therapeutic window of this drug combination. Indeed, the value of this approach has already been shown by recent studies (*e.g.*,^30,31,59–61^). As the evaluation of ligand release kinetics in living cells is challenging, we also wanted to have a tool to visualize this process. Therefore, we chose the EGFR inhibitor KP2187, exhibiting strong intrinsic fluorescent properties and coupled it to an oxaliplatin(iv) core. Indeed, the novel platinum-EGFR inhibitor complex KP2749 exhibited quenched fluorescent properties, which allowed us to study the release of KP2187 by confocal microscopy in living cancer cells over time. Together with the subsequently collected cell and molecular biological data, we uncovered that the intact KP2749 is already a potent EGFR inhibitor, which releases oxaliplatin in the second phase of its activity. Interestingly, also in line with reports on another EGFR-TKI-releasing platinum(iv) complex,^31^ the complexation of the platinum core to an EGFR-inhibitory moiety resulted in distinctly superior drug uptake *via* an active transport mechanism. The exact nature of this mechanism is currently unknown. However, the EGFR is frequently internalized *via* endocytosis.^62^ Consequently, it can be hypothesized that bound KP2749 is taken up in course of this process. Noteworthy, *in silico* docking studies showed that the binding to the EGFR is different between KP2187 and KP2749, indicating that coupling to metal cores such as platinum can be used to fine-tune EGFR inhibitors, a strategy which can probably also be expanded to other tyrosine kinase inhibitors. For example, there are some platinum(iv) complexes with imatinib, nilotinib or dasatinib as ligands.^52,63^

Consequently, this study presents not only a novel multi-action platinum(iv) complex with the ability to overcome both oxaliplatin and EGFR-TKI resistance in cell culture and in mouse models but in turn also a new tool, which can be used for the imaging of ligand release from platinum(iv) complexes in living cells.

## Materials and methods

All information regarding the synthesis, chemical characterization, experimental details and used materials for this article have been included as part of the ESI.†

## Author contributions

Conceptualization: MC, PF, AT, JHBN, CRK, PH; data curation: MC, PF, AT, RP, DK, CRK, PH; formal analysis: MC, PF, AT, RP, CRK, PH; funding acquisition: JHBN, WB, BKK, CRK, PH; investigation: MC, PF, AT, AF, RP, DK, HHS, PH; methodology: MC, PF, AT, DK, CRK; project administration: JHBN, CRK, PH; resources: WB, BKK, CRK, PH; supervision: JHBN, HHS, WB, BKK, CRK, PH; validation: all authors; visualization: MC, PF, AT, DK, CRK, PH; writing – original draft: MC, PF, AT, CRK, PH; writing – review & editing: all authors.

## Data availability

The data supporting this article have been included as part of the ESI.†

## Conflicts of interest

There are no conflicts to declare.

## Supplementary Material

QI-012-D4QI03025G-s001

QI-012-D4QI03025G-s002
