# Supplementary material for: A new fluorescent oxaliplatin(iv) complex with EGFR-inhibiting properties for the treatment of drug-resistant cancer cells
Source: Inorg Chem Front. 2025 Jan 9;12(4):1538–52. doi: 10.1039/d4qi03025g (PMC11715172; doi:10.1039/d4qi03025g)
Supplement: QI-012-D4QI03025G-s001 [file QI-012-D4QI03025G-s001.pdf]

Supplementary Information (SI) for Inorganic Chemistry Frontiers.
This journal is © the Partner Organisations 2024

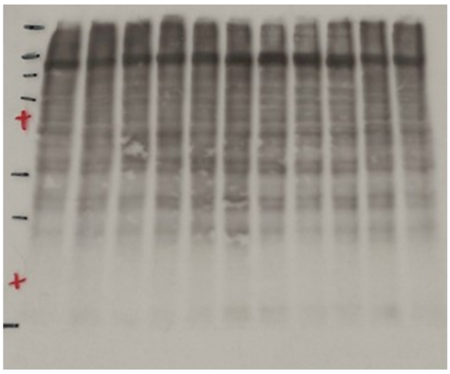

EGFR

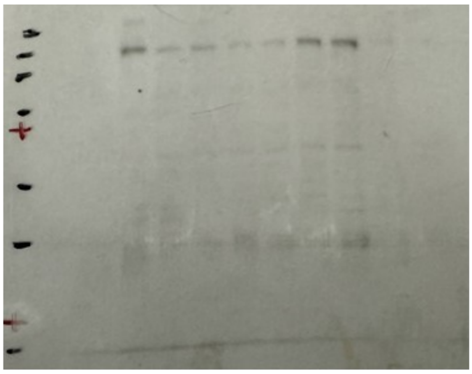

p- EGFR

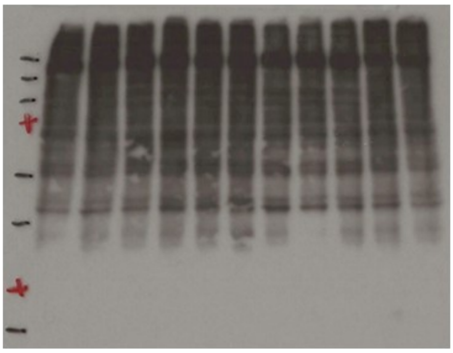

ERK 1/2

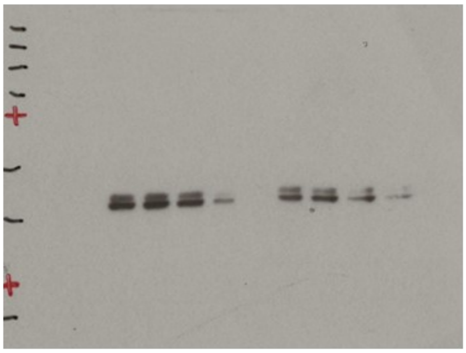

p- ERK 1/2

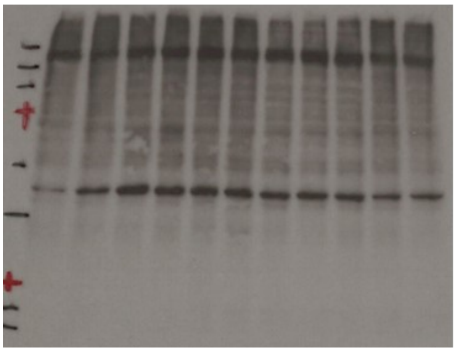

β - Actin

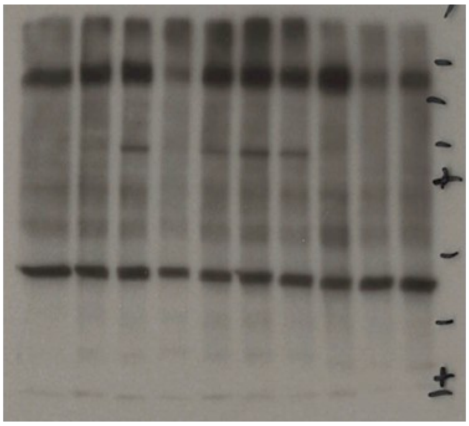

EGFR

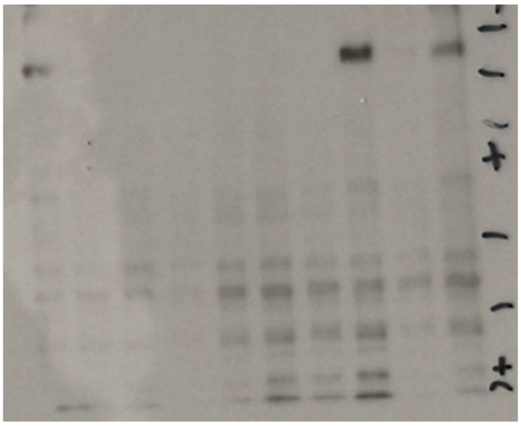

p- EGFR

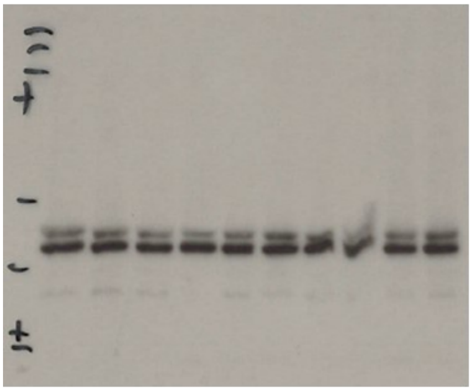

ERK 1/2

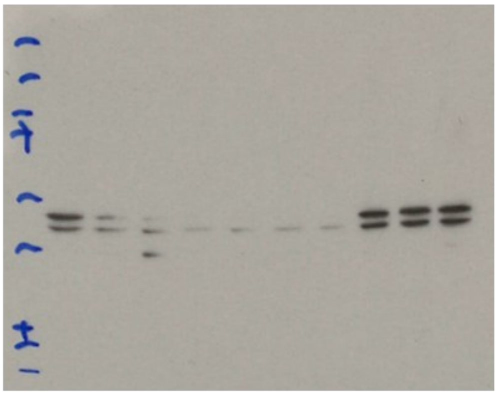

p- ERK 1/2

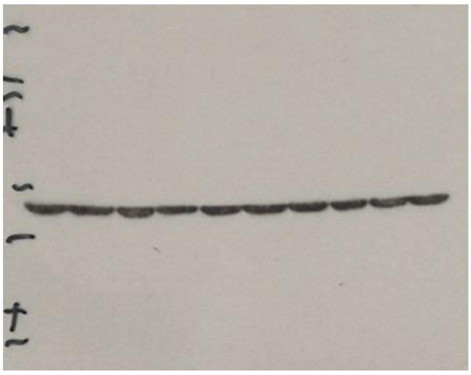

β - Actin

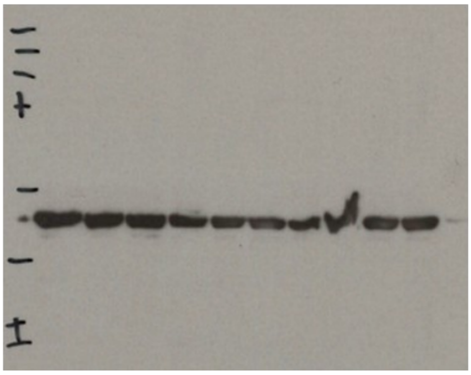

β - Actin

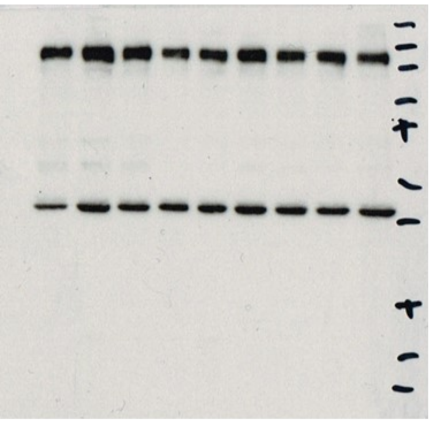

EGFR

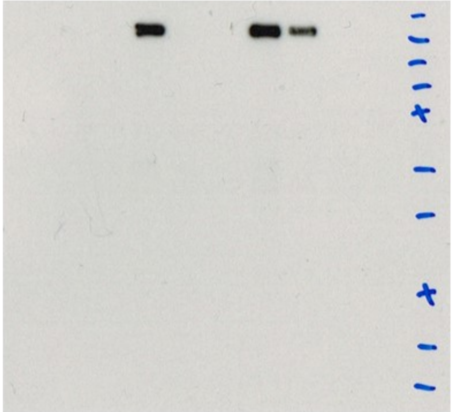

p- EGFR

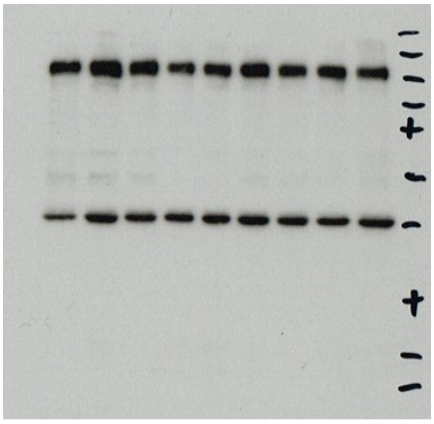

ERK 1/2

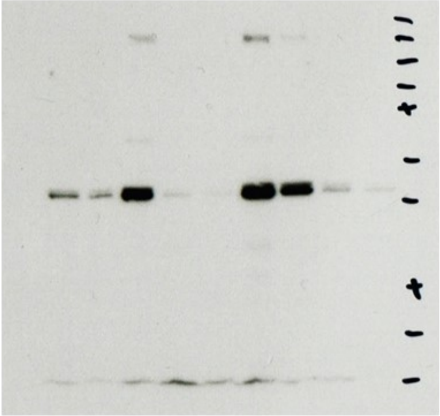

p- ERK 1/2

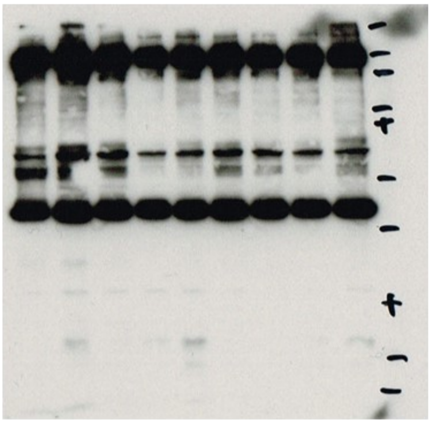

AKT

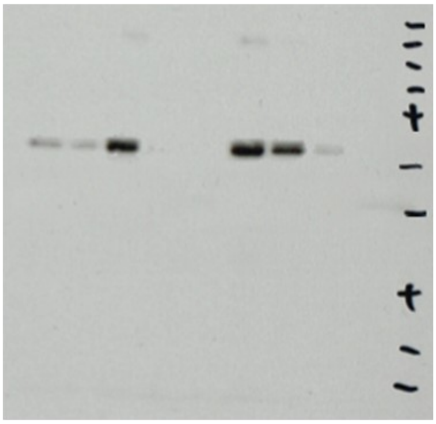

p-AKT

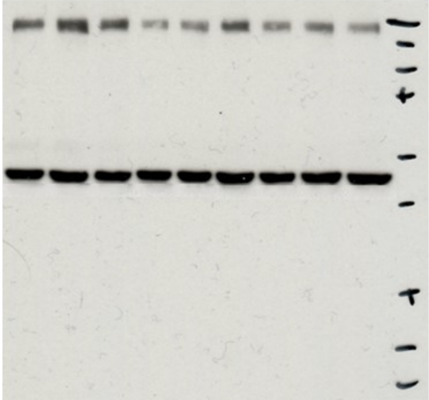

β - Actin
