# Supplementary material for: A new fluorescent oxaliplatin(iv) complex with EGFR-inhibiting properties for the treatment of drug-resistant cancer cells
Source: Inorg Chem Front. 2025 Jan 9;12(4):1538–52. doi: 10.1039/d4qi03025g (PMC11715172; doi:10.1039/d4qi03025g)
Supplement: QI-012-D4QI03025G-s002 [file QI-012-D4QI03025G-s002.pdf]

Supplementary Information (SI) for Inorganic Chemistry Frontiers.
This journal is © the Partner Organisations 2024

Supplementary Information

A new fluorescent oxaliplatin(IV) complex with EGFR-inhibiting properties for the treatment of drug-resistant cancer cells

Monika Caban[1§], Philipp Fronik[2§], Alessio Terenzi[3], Anja Federa[2,4], Julia H. Bormio Nunes[1,2], Rastislav Pitek[1], Dominik Kirchhofer[1], Hemma H. Schueffl[1], Walter Berger[1,5], Bernhard K. Keppler[2,5], Christian R. Kowol[2,5*], Petra Heffeter[1,5*]

[1] Center for Cancer Research and Comprehensive Cancer Center, Medical University of Vienna, Austria;

[2] Institute of Inorganic Chemistry, Faculty of Chemistry, University of Vienna, Austria;

[3] Department of Biological, Chemical and Pharmaceutical Sciences, University of Palermo, Viale delle Scienze, Ed. 17, 90128, Palermo, Italy

[4] Research Cluster "Translational Cancer Therapy Research", University of Vienna and Medical University of Vienna, Austria

[§] these authors contributed equally to the main findings of the manuscript

Material and methods

Chemicals. Potassium tetrachloridoplatinate (K_2PtCl_4) was purchased from Johnson Matthey (Switzerland). Water for synthesis was taken from a reverse osmosis system and distilled twice before use. For HPLC measurements Milli-Q water (18.2 MΩ·cm, Merck Milli-Q Advantage, Darmstadt, Germany) was used. Chemicals and solvents were purchased from commercial suppliers (Sigma Aldrich, Merck, Acros, Fluka and Fisher Scientific). Oxaliplatin and the respective platinum(IV) complex OxOH/OAc were synthesized similarly to methods described in literature [57, 58]. KP2187 was synthesized as previously described [36]. Electrospray ionization (ESI) mass spectra were recorded on a Bruker amaZon SL ion trap mass spectrometer in positive and/or negative mode by direct infusion at the Mass Spectrometry Centre of the University of Vienna. One- and two-dimensional ^{1}H-, ^{13}C-, and ^{19}F-NMR spectra were recorded on a Bruker AV Neo 500 or AV III 600 spectrometer at 298 K. For ^{1}H- and ^{13}C-NMR spectra the solvent residual peak was taken as internal reference. The ^{1}H- and ^{13}C-NMR spectra of complex KP2749 as well as the NMR numbering scheme are depicted in Suppl. Figure S8. Purification by preparative reverse-phase HPLC was performed on an Agilent 1200 series system using a Waters XBridge C18 column (19x250 mm). Elemental analysis measurements were done on a Perkin Elmer 2400 CHN Elemental Analyzer at the Microanalytical Laboratory of the University of Vienna and are within ±0.4%, confirming >95% purity. Furthermore, a UPLC chromatogram of KP2749 can be found in Suppl. Figure S9. Erlotinib, gefitinib and oxaliplatin for biological assays were purchased from LC laboratories. For cell culture studies erlotinib and gefitinib were dissolved in dimethyl sulfoxide (DMSO, 10 mM stocks), oxaliplatin was dissolved in bidest. water and the synthesized compounds were dissolved either in DMSO (KP2187) or in DMF (KP2749) and stocks stored at –20°C. For the

experiments, stocks were diluted in cell culture medium (DMSO as well as DMF concentrations were always <1%).

Synthesis

(*OC*-6-34)-Acetato[(2-((4-((3-bromophenyl)amino)quinazolin-6yl)amino)ethyl)carbamato] [(1*R*,2*R*)-1,2-cyclohexanediamino] oxalatoplatinum(IV) trifluoroacetate (KP2749). KP2187 (present as two-fold HCl salt [36]) was dissolved in 1 M NaOH and extracted three times with EtOAc. The organic layer was washed with brine, dried over Na_2SO_4 and evaporated to dryness in order to obtain the free base as a yellow solid. In a dry round bottom flask Ox(OH)(OAc) (200 mg, 0.42 mmol) was dissolved in anhydrous DMF (7 mL) and *N*,*N*'-disuccinimidyl carbonate (141 mg, 0.55 mmol, 1.3 equiv.) was added and stirred for 3 h at room temperature (rt) under Ar. The free base of KP2187 (242 mg, 0.68 mmol, 1.6 equiv.) was added and the reaction mixture stirred for 18 h at rt under Ar. The solvent was removed *in vacuo* and the crude product purified by preparative RP-HPLC (25-40% MeCN (containing 0.1% Trifluoressigsäure (TFA) in H_2O (containing 0.1% TFA); flow rate 25 mL/min) to obtain (*OC*-6-34)-acetato[(2-((4-((3-bromophenyl)amino)quinazolin-6-yl)amino)ethyl)carbamato][(1*R*,2*R*)-1,2-cyclohexanediamino]oxalatoplatinum(IV) trifluoroacetate (175 mg, 39 %) as a bright yellow solid.

^{1}H NMR (700 MHz, DMSO-d_6)δ 10.70 (bs, 1H, E-11), 9.60 (b s, 1H. D-1), 8.79 – 8.44 (m, 2H, E-8, D-1), 8.44 – 8.15 (m, 2H, 2x D-1), 8.09 – 7.98 (m, 1H, E-13), 7.74 (d, *J* = 7.6 Hz, 1H, E-17), 7.64 (d, *J* = 8.9 Hz, 1H, E-3), 7.57 – 7.38 (m, 3H, E-2, E-15, E-16), 7.38 – 7.14 (m, 1H, E-6), 6.98 – 6.46 (m, 2H, E-18, E-21), 3.26 – 3.07 (m, 4H, E-19, E-20), 2.58 – 2.56 (m, 2H, D-2), 2.21 – 2.05 (m, 2H, 2x D-3), 2.03 – 1.92 (m, 3H, acetate), 1.56 – 1.39 (m, 3H, 2x D-4, 1x D-3), 1.39 – 1.31 (m, 1H, D-3), 1.23 – 1.04 (m, 2H, 2x D-4); ^{13}C NMR (176 MHz, DMSO) δ 178.3 (acetate-C=O), 164.4

(E-22), 163.5, 163.4 (2x oxalate-C=O), 157.7 (E-10), 149.0 (E-1), 146.8 (E-8), 139.1 (E-12), 130.7 (E-16), 128.4 (E-15), 126.6 (E-13), 125.9 (E-2), 123.0 (E-17), 122.0 (E-3, detected via HSQC) 121.2 (E-14), 115.5 (E-5), 97.4 (E-6), 61.2, 60.8 (2x D-2), 43.6, 43.3 (2x E-20), 40.0 (E-19, detected via HSQC) 31.0, 30.8 (2x D-3), 23.6, 23.4 (2x D-4), 22.9 (acetate-CH_3). "D" stands for "diaminocyclohexane" and "E" for the "EGFR inhibitor"-ligand. The numbering scheme can be found in Suppl. Figure S8; MS (m/z): calcd. $C_{27}H_{31}N_7O_8BrPt$, $[M - H^+]^-$, 856.10; found, 856.07. EA: calcd. $C_{29}H_{33}N_7O_{10}BrF_3Pt$*0.5TFA*0.5$H_2O$, C: 34.73, H: 3.35, N: 9.45; found, 34.83, H: 3.34, N: 9.46.

UPLC/LCMS measurements. KP2749 was dissolved in 10% DMF in PB (500 mM, pH 7.4) to a final concentration of 500 µM with or without the presence of 5 mM AA. The samples were incubated at the desired temperature and measured on either a Dionex UltiMate 3000 RS UPLC system equipped with a Waters Acquity UPLC® BEH C18 column (3x50 mm, pore size 1.7 µm) and absorption detection at 220 nm or an Agilent 1260 Infinity system using a Waters Atlantis T3 column 150 mm x 4.6 mm coupled to a Bruker amaZon SL ESI-IT mass spectrometer.

Log $D_{7.4}$ determination. A 5 mM stock solution of KP2749 (in DMF) was diluted in PB (20 mM, pH 7.4, pre-saturated with n-octanol) to a final concentration of 50 µM drug and 1% DMF. Likewise, oxaliplatin was dissolved in the same PB to the same final concentrations and 1% DMF was added. The platinum content of these aqueous solutions was measured by ICP−MS (see below). The solutions were mixed 1:1 with n-octanol and shaken on a 360° rotatable rack for 3 h. The solutions were centrifuged for 5 min (860 g, RT), the aqueous layers were carefully removed, and their Pt content was measured by ICP−MS. Experiments were performed and measured in triplicates, and averages were used for further calculations. log $D_{7.4}$ values were calculated by the following equation:

$$\log D_{7.4} = \frac{[c(Pt\ aqueous\ stock) - c(Pt\ aqueous\ layer)]}{c(Pt\ aqueous\ layer)}$$

Molecular docking. AutoDock 4.2 was used to perform molecular docking [59]. The Protein Data Bank file PDBid: 4G5J was used as model for EGFR (wild type) in complex with afatinib while PDBid: 5CAS was used as model for EGFR kinase domain mutant "TMLR". Autodock Tools 1.5.6 software was used to prepare both receptors and ligands. The structures of KP2187 and KP2749 were optimized by DFT calculations using Gaussian16 [60]. For these calculations, LANL2DZ was used as the basis set for Pt [61], while the 6-31G* basis set was employed for the other atoms [62].

To perform the docking calculations, we created a grid box that adequately covered the protein's binding pocket along with potential ligand-receptor complexes. More precisely, the grid dimensions were set to 100 × 100 × 100 points, with a grid spacing of 0.375 Å. The grid centers were determined based on the center of mass of the original ligands as found in the PDB databank. In detail, coordinates x= 50.68, y= 1.374, and z= -21.036 were used for PDBid: 4G5J, and x= -51.907, y= -0.508, and z= -23.173 for PDBid: 5CAS. The Lamarckian Genetic Algorithm was used for the docking process, with default parameter settings. The estimated free energies of binding were expressed in kcal/mol. Biovia Discovery Studio Visualizer v24.1 (San Diego) was used to generate 2D interaction diagrams, while Chimera X [63], was used to visualize the docking results. Wild type 4G5J and mutated 5CAS EGFR were aligned and superimposed using the Chimera X tool “MatchMaker” in order to better compare the binding of the selected molecules with the targets.

Cell culture. Detailed information about the used cell lines is provided in Supplementary Table S1. All cell cultures were grown under standard conditions and regularly checked for

Mycoplasma contamination. Fetal bovine calf serum (FBS) was purchased from PAA (Linz, Austria). The HCT116/OxR as well as the TKI-resistant HCC827 cell models were continuously selected with the indicated drugs.

Cell viability assay. Cells were plated (depending on the cell model 2-4 x 10^4 cells/mL) in 96-well microtiter plates and allowed to recover for 24 h. Subsequently, the indicated drugs were added. After 72 h exposure, the cell viability was determined by an MTT-based cell viability assay following the manufacturer's recommendations (EZ4U kit, Biomedica). Cytotoxicity was expressed as IC_{50} values calculated from full dose-response curves using GraphPad Prism 8 software. All experiments were performed in triplicates.

Clonogenicity assay. Cells were plated in 24-well plates (2-3 x 10^3 in 500 µL growth medium per well) and left to recover for 24 h in the incubator. Then the cells were treated with prediluted ascending concentrations of the tested compounds. After 10 days, the cells were fixed with methanol for 20 min at 4°C and stained with crystal violet (0.01% in phosphate-buffered saline (PBS)). The fluorescence of crystal violet was detected with the Typhoon scanner (Typhoon TRIO Variable Mode Imager, GE Healthcare Life Sciences) after excitation by the red laser (633 nm). Quantification was performed using ImageJ software. The received values were visualized as dose-response curves by Graph Pad Prism 8 software (Graph Pad software, USA).

Cellular platinum uptake determined by ICP-MS. Cells were seeded at a concentration of about 1 x 10^6 cells into 6-well plates in triplicates plus two additional wells for each condition for cell counting. Cells were left overnight to recover following 5 h treatment with 1 mL of 10 µM drug solution of each of the tested drugs. For each condition, three blank wells with no cells were treated with the same drug concentrations. All wells (cells, blanks and wells for

counting) were washed twice with PBS. The wells for counting were trypsinized and the total cell amount calculated. To lyse the cells and prepare the samples for ICP-MS measurement, 500 µL of 67% HNO_3 were added to each sample and incubated for 1 h at rt. From each well 400 µL were transferred into a falcon tube containing 7.6 mL H_2O. The Pt content (µg/l) of each sample was measured by ICP-MS and the final Pt concentration calculated and normalized to the cell amount (ng/ 10^6cells). Statistical analysis was done by two-way-ANOVA with Bonferroni correction. The measurements were performed on an Agilent 7800 ICP-QMS instrument (Agilent Technologies, Tokyo, Japan) equipped with an Agilent SPS 4 autosampler (Agilent Technologies, Tokyo, Japan) and a MicroMist nebulizer at a sample uptake rate of approx. 0.2 mL/min. The Agilent MassHunter software package (Workstation Software, Version C.01.04, 2018) was used for data evaluation. All measured samples were blank corrected (for the cell culture data, wells containing no cells were used). The instrumental parameters for the ICP−MS are summarized in Suppl. Table S2. Elemental standard solutions were purchased from Labkings (Hilversum, The Netherlands). The instrument was tuned daily.

Western blot analysis. To investigate the impact of the drugs on the EGFR signaling pathway and further downstream effectors, A431 cells were starved by serum deprivation for 24 h before treatment to reduce the impact of other tyrosine kinase receptors. Then cells were incubated with the ascending concentrations of either KP2187 or KP2749 for 4 h (0.5, 1, 2.5 and 5 µM). To assure EGF-dependent activation of the signaling pathway, EGFR stimulation was induced 10 min before protein isolation by adding 50 ng/mL EGF. For H1650 cells no EGF stimulation was necessary and cells were treated with the ascending concentrations of the above drugs for 24 h. The total protein lysates were transferred onto a polyvinylidene difluoride membrane and examined via Western blot analysis. Primary antibodies for EGFR

(clone D38B1), pEGFR (clone D7A5), ERK1/2 (clone 137F5) and pERK (clone D13.14.4E) (p44/p22), AKT(Pan) and p-AKT were provided by *Cell signaling* and used at a 1:1000 dilution (in 3% bovine serum albumin (BSA)). Secondary antibodies were conjugated to horseradish peroxidase and were used at a 1:10.000 dilution (in 1% BSA).

Cell-free fluorescence measurement of KP2187 and KP2749. Both compounds were diluted to a final concentration of 10 µM in cell culture medium and plated onto a black bottom 96-well-plate in duplicates. Fluorescence intensity was determined using a Tecan reader at 370 nm. Measured values were normalized to blank values and analyzed using GraphPad Prism 8.

Immunofluorescence and imaging

KP2187-ligand release. H1650 cells were seeded 8 x 10^4 cells/mL 100 µL/well on µ-96-well black polystyrene imaging plates (Perkin Elmer) with optical clear bottom and treated with 5 µM of either KP2749 or KP2187. The investigated compounds were spiked directly into each well before imaging at different time points starting from 24 h, 5 h, 4 h, 3 h, 2 h, 1 h, 30 min and 10 min. Automated wide-field microscopy for QIBC was performed on an Olympus IXplore SpinSR inverted research microscope using 40X magnification (Olympus, Life Science Solutions). 9-images/well were taken of each well. A neuronal network was trained for label-free cell detection using the Olympus cellSens Dimension Desktop 4.2 software Deep learning package and applied on all the images. Images were analyzed with the Olympus ScanR Image Analysis Software (Olympus OSIS Life Science Solutions, version 3.3). Blue-fluorescent signal of the compounds was quantified within the segmented cells and results depicted using GraphPad Prism 8.

pH2aX staining and quantification. H1650 cells were seeded 8 x 10^4 cells/mL 100 µL/well on µ-96-well black polystyrene imaging plates (Perkin Elmer) with clear optical bottom and treated with increasing concentrations of either KP2749, KP2187 or oxaliplatin for 24 h in triplicates. Cells were pre-extracted with 0.1% Tween in PBS for 1 min at rt after medium removal, fixed with 4% PFA for 20 min at rt and permeabilized using 0.5% Triton (in PBS) for 10 min at rt. After blocking with 5% BSA cells were labeled with anti-phospho-histone H2A.X (Ser139) (D7T2V) Mouse mAb #80312 primary antibody; 1:400, cell signaling overnight at 4°C. Cells were washed three times with 3% BSA in PBS plus one time with PBS only and incubated with Alexa Fluor 488 fluorescence-labelled anti-mouse IgG secondary antibody (Cell Signaling) for 1 h at rt. Nuclear staining with 1 µg/mL DAPI (1: 1000 in PBS) for 10 min at rt was performed for easier cell detection. Automated multichannel wide-field microscopy for quantitative image-based cytometry (QIBC) was performed on an Olympus IXplore SpinSR inverted research microscope using 40X magnification (Evident, Life Science Solutions). Images were analyzed with the Olympus ScanR Image Analysis Software (Olympus OSIS Life Science Solutions, version 3.3), nuclei segmentation was performed using a neuronal network-based object detection module based on the DAPI signal. Mean intensities were quantified and the increase in pH2aX-signal depicted as pH2aX positive nuclei normalized to the untreated controls.

Determination of full fluorescent spectrum of KP2187 and KP2749 using Cytek Aurora Full Spectrum Flow Cytometer. H1650 cells were harvested, resuspended in FACS-PBS and transferred into 1 mL FACS tubes. After pre-incubation with the tested compounds for 1 h, samples were measured, and fluorescence spectrum was determined using a Cytek Aurora (CYTEK®) equipped with 5 lasers (range from 365-829 nm) and 64 detectors. Fluorescence Raw

Data was unmixed using SpectroFlo Unmixing Algorithm (Brand Name). Cell populations were compared using MFI.

Animals. Eight-to-twelve weeks-old C.B.17 SCID mice were purchased from Envigo, Italy. The animals were kept in a pathogen-free environment and every procedure was done in a laminar airflow cabinet. Experiments were done according to the regulations of the Ethics Committee for the Care and Use of Laboratory Animals at the Medical University Vienna (BMBWF-66.009/0394-V/3b/2018), the U.S. Public Health Service Policy on Human Care and Use of Laboratory Animals as well as the United Kingdom Coordinating Committee on Cancer Prevention Research's Guidelines for the Welfare of Animals in Experimental Neoplasia. To ensure animal welfare throughout the experiment, the body weight of the mice was assessed once daily.

Therapy experiment in H1650 and H1975 xenografts. For therapy experiments, either H1650 or H1975 cells (1×10^6 in 50 µL) were injected subcutaneously into the right flank. Animals were randomly assigned to treatment groups and therapy was started when tumor nodules were about 175 mm^3. Animals were treated i.p. with the indicated drugs twice per week for two weeks with oxaliplatin (dissolved in 5% glucose), or three times per week for two weeks in case of KP2749 (49 mg/kg dissolved in 25% propylene glycol in 0.9% NaCl) and KP2187 (20 mg/kg dissolved in 0.9% NaCl). Animals in the control group received 25% PG in 0.9% NaCl only. The condition and behavior of the animals were controlled every day and tumor size was assessed regularly by caliper measurement. Tumor volume was calculated using the formula: $(\text{length} \times \text{width}^2) / 2$. Endpoint of the experiment was overall survival. Most of the animals were sacrificed due to ulceration at a tumor size of ~1000 mm^3. For histological and immunohistochemical analysis of H1650 tumor tissue, a second experiment with the same

settings was carried out and all mice were sacrificed after end of therapy. The collected tumors were formalin-fixed and paraffin-embedded.

Short-term therapy experiment in A431 xenografts. A431 cells (1×10^6 in 50 µL) were injected subcutaneously into the right flank (n=2). When the tumor reached a size of 200-250 mm^3 the animals were treated i.p. with a single dose of either KP2187 (9.8 mg/kg) or KP2749 (23.5 mg/kg) for 24 h. After therapy all mice were sacrificed and the tumors collected for immunohistochemical analysis.

Immunohistochemistry. Fresh sections from paraffin-embedded A431 and H1650 tumors were deparaffinized and dehydrated. In brief, after antigen retrieval by boiling for 30 min in 10 mM citrate buffer (pH 6.0), sections were incubated with pEGFR-specific antibody (Tyr1068, Cell signaling, 1:200) in a humid chamber for 1 h at rt. Antibody binding was detected using the UltraVision LP detection system according to the manufacturer's instructions (Thermo Fisher Scientific Inc.). Color was developed using 3,3′-diaminobenzidine (DAB; Dako). Stained slides were scanned with a Slide Scanner (3DHISTECH Pannoramic Scan II), together with a DS-U3 control unit and the adequate NIS-Elements software (all from Nikon Instruments). Evaluation and quantification of the staining was done by HALO software.

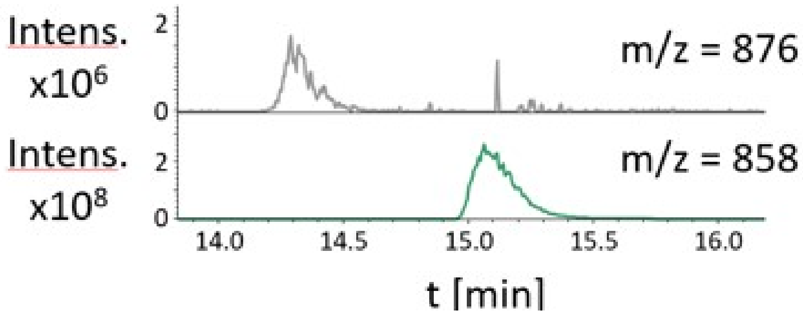

Suppl. Figure S1. Extracted LC-MS ion chromatograms of KP2749 (m/z = 858) and its mono-hydrolyzed form (m/z = 876) after 24 h incubation in PB at 20°C without AA.

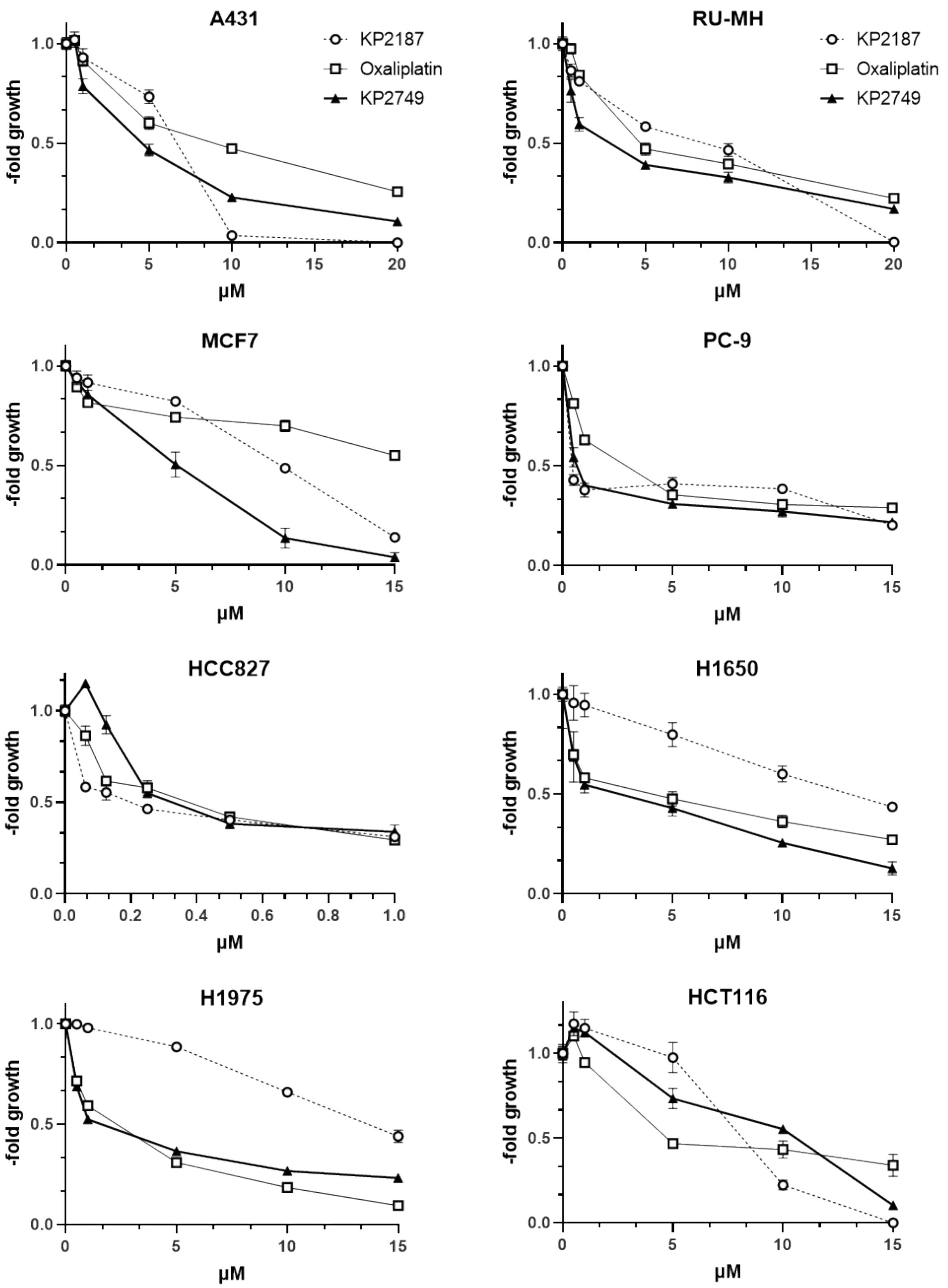

Suppl. Figure S2. Cytotoxic activity of KP2749 compared to KP2187 and oxaliplatin in different cell lines. The cells were incubated with the indicated drugs for 72 h. Cell viability was measured using an MTT-based assay. Values are given as means ± SD of one representative experiment performed in triplicates.

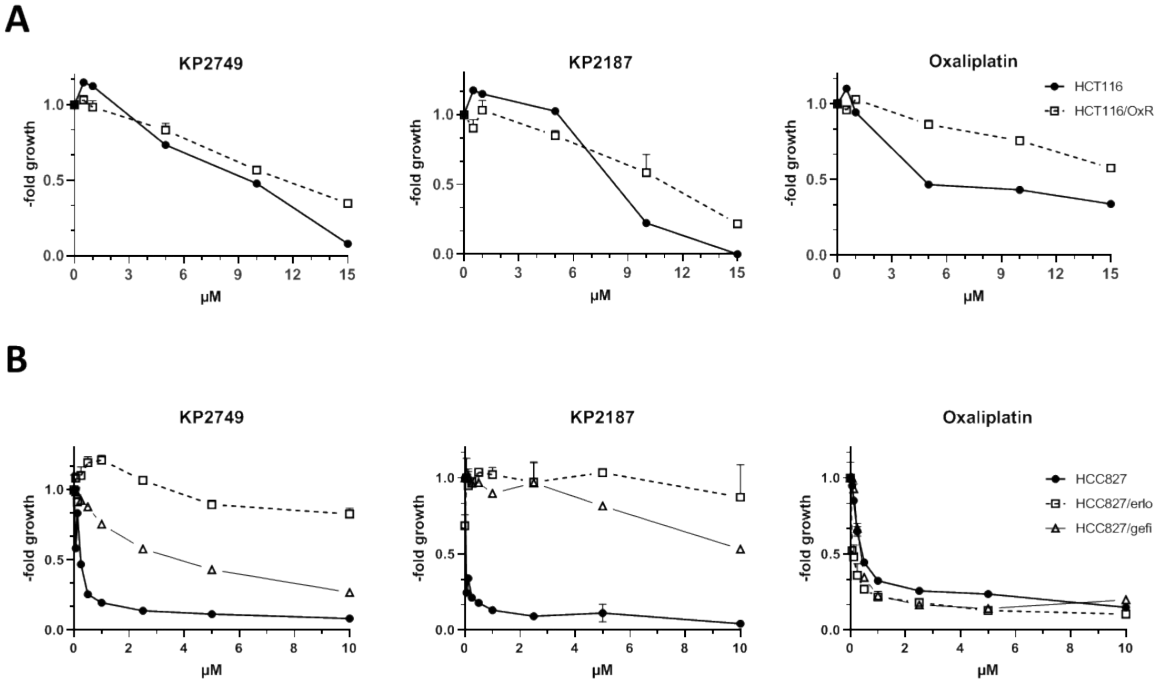

Suppl. Figure S3. Cytotoxic activity of KP2749 vs. KP2187 and oxaliplatin in an oxaliplatin-resistant cell model HTC116 (A) as well as in the EGFR TKI-resistant cell models HCC827/Erlo and HCC827/Gefi (B). The cells were incubated with the indicated drugs for 72 h. Cell viability was measured using an MTT-based assay. The values in the dose-response curves are given as means ± SD of one representative experiment performed in triplicates.

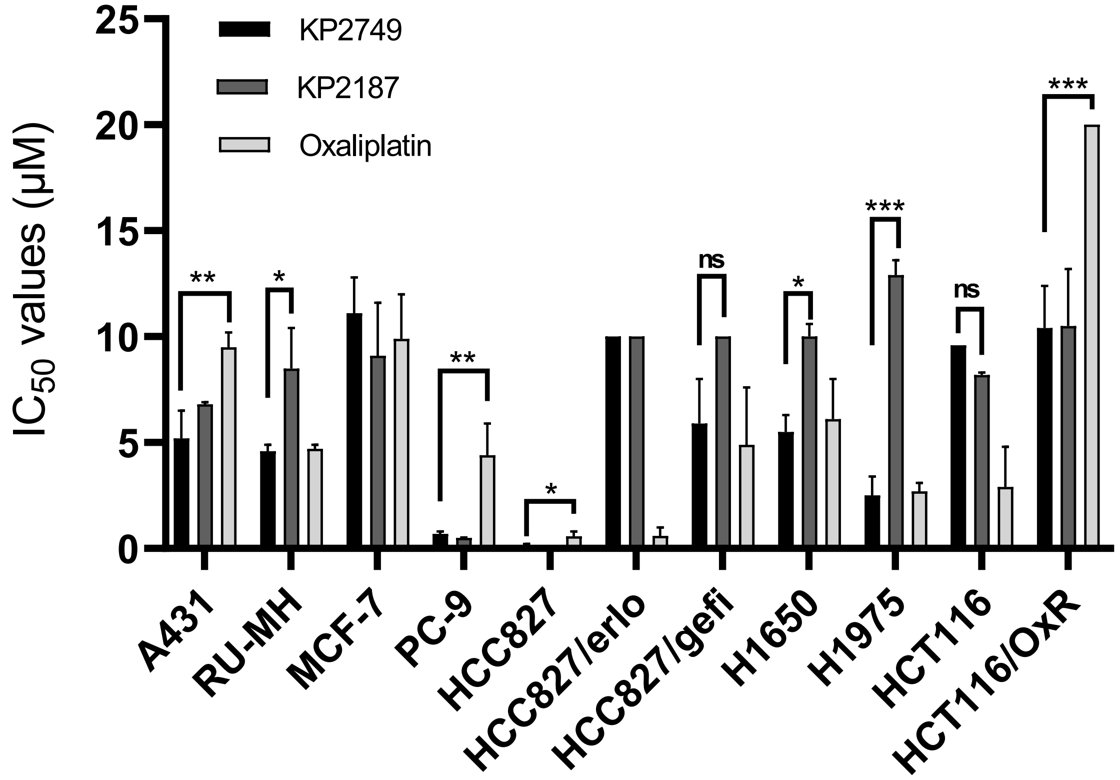

Suppl. Figure S4. IC_{50} values of KP2749, KP2187 and oxaliplatin. Bar graph of the of IC_{50} values (in µM) listed in Table 1 showing the statistical differences in activity of either KP2749 vs KP2187 or KP2749 vs oxaliplatin. Statistical significance was tested by ordinary one-way ANOVA and Tukey's multiple comparison Test (*p < 0.05, **p < 0.01 and ***p < 0.001)

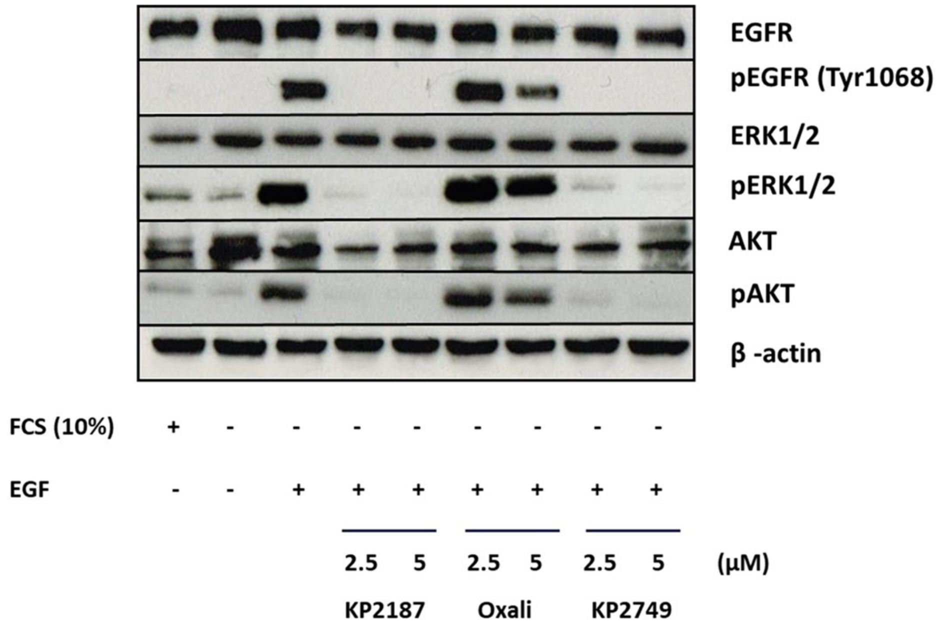

Suppl. Figure S5. Inhibition of EGFR phosphorylation in cell culture. Western blot analysis of total protein lysates of A431 cells are shown. The cells were cultured with (+) or without (-) 10% FCS and treated for 4 h with the indicated concentrations of KP2187, oxaliplatin, and KP2749. The EGFR was stimulated for 10 min with 50 ng/mL EGF (+). Equal amounts of cellular proteins (15 µg/lane) were loaded. EGFR, ERK1/2 and AKT as well as the respective phosphorylated forms were investigated. β-actin served as loading control.

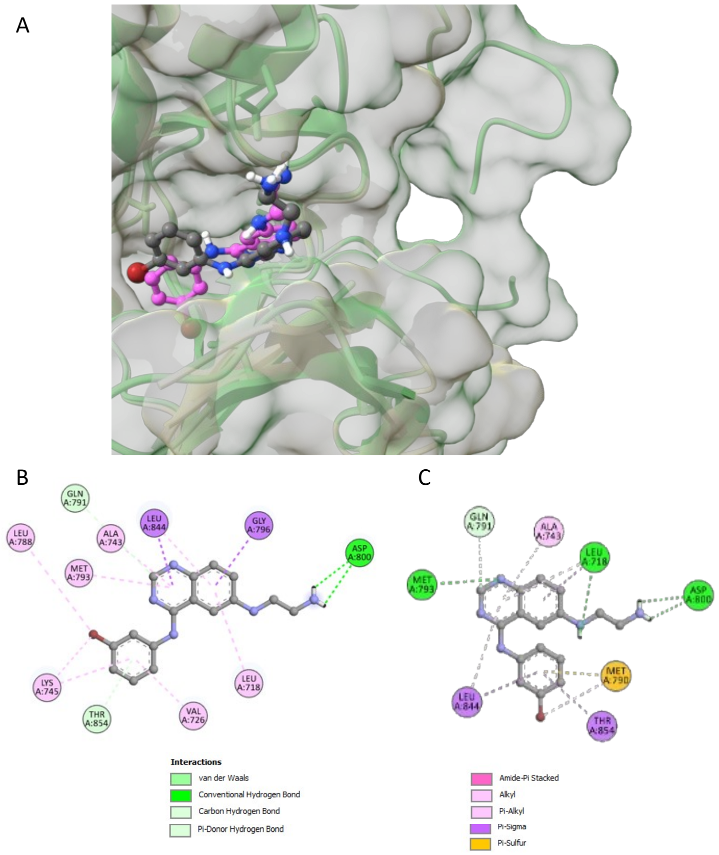

Suppl. Figure S6. *In silico* docking studies of the compounds to the EGFR molecules. (A) 3D representation of EGFR/wt (PDBid: 4G5J, gold surface) interacting with KP2187 (in pink) aligned with mutant EGFR (PDBid: 5CAS, green surface) bound to KP2187 (in dark grey). Results were obtained by molecular docking performed using AutoDock. (B, C) Schematic 2D diagrams build with Biovia Discovey Studio Visualizer of protein–ligand interactions for KP2187 in complex with wild-type (B) and mutant (C) EGFR. Interaction types are depicted in the legend.

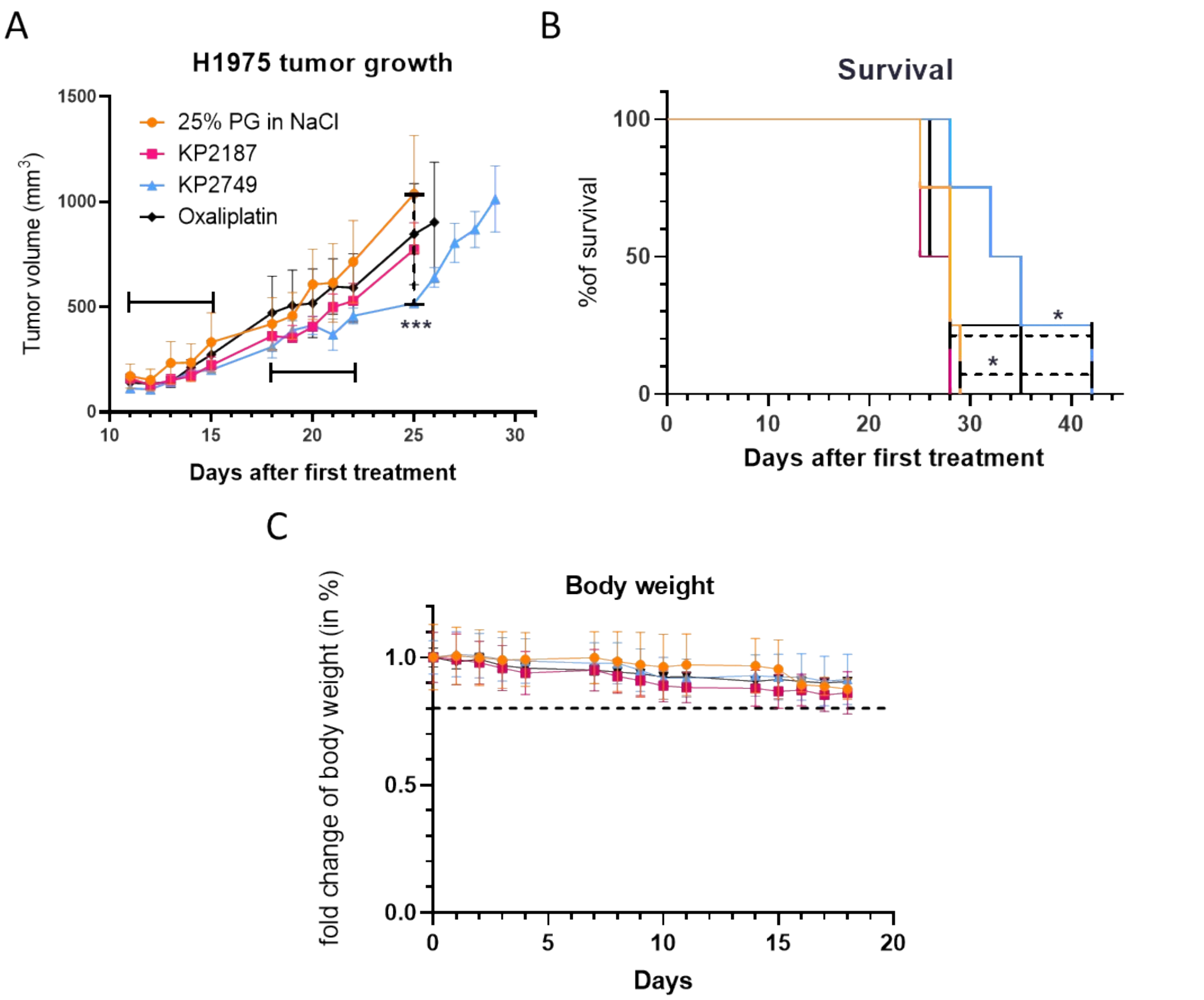

Suppl. Figure S7. H1975 cells were injected s.c. into the right flank of male C.B.-17/SCID mice. When the tumors were ~175 mm^3 in size, the animals were treated i.p. with the indicated drugs for two weeks either twice per week with oxaliplatin (6 mg/kg) or three times per week with KP2749 (49 mg/kg) and KP2187 (20 mg/kg). (A) Impact on tumor growth; data are presented as means ± SEM. (B) The overall survival of the animals is depicted via a Kaplan–Meier curve. Statistical significance was tested by log-rank test and Mantel–Cox posttest. (C) The body weight was monitored throughout the whole experiment and measured on 5 consecutive days until mice were sacrificed.

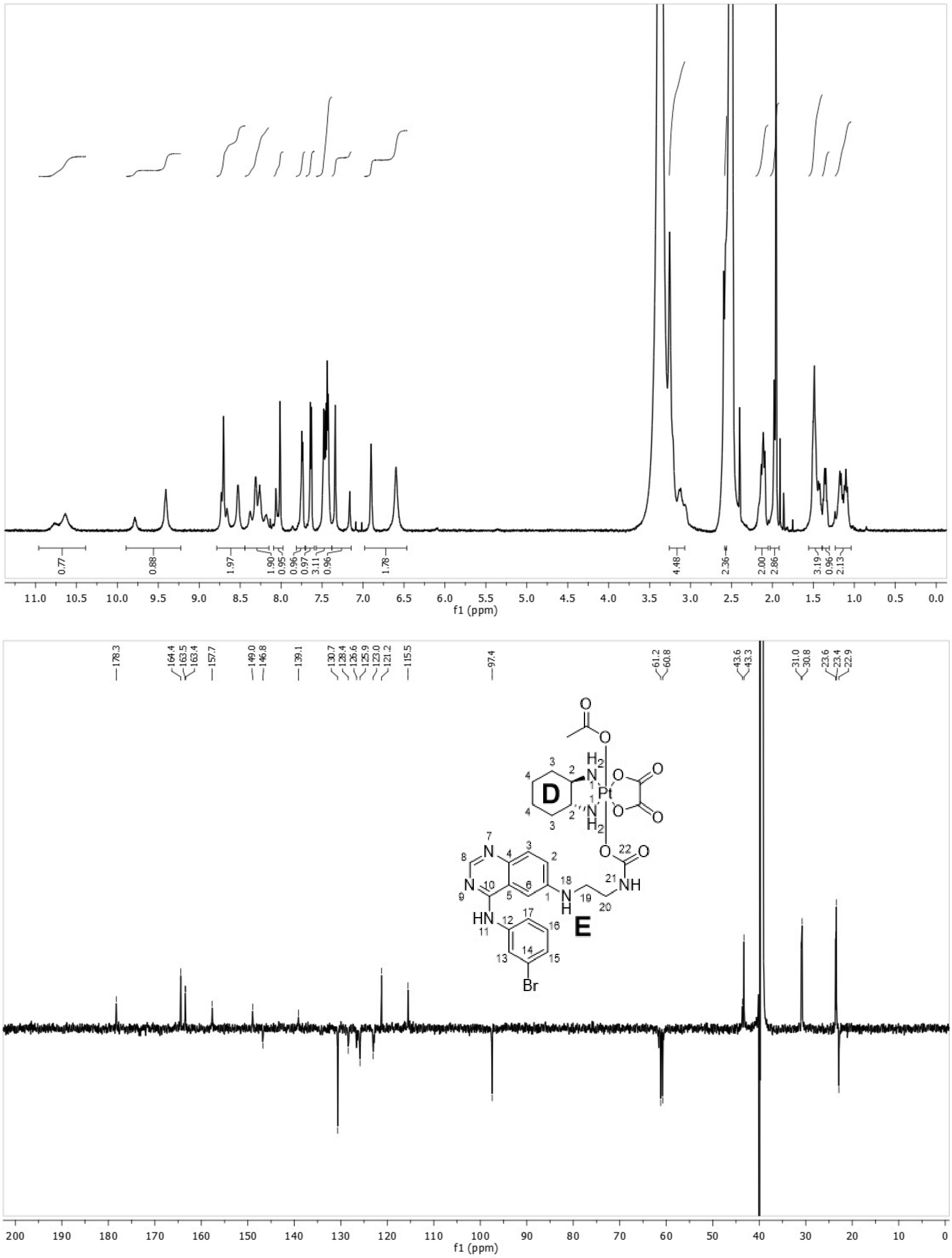

Suppl. Figure S8. ^{1}H and ^{13}C NMR spectra, as well as the numbering scheme for the 1,1-diaminocyclohexane (DACH, D) and the EGFR-inhibitor ligand (E) of complex KP2749. In the ^{1}H spectrum, the peaks in the range of 11.0-9.3 ppm as well as between 7.5 and 6.5 ppm are split up due to the different orientations of the carbamate moiety at the platinum core.

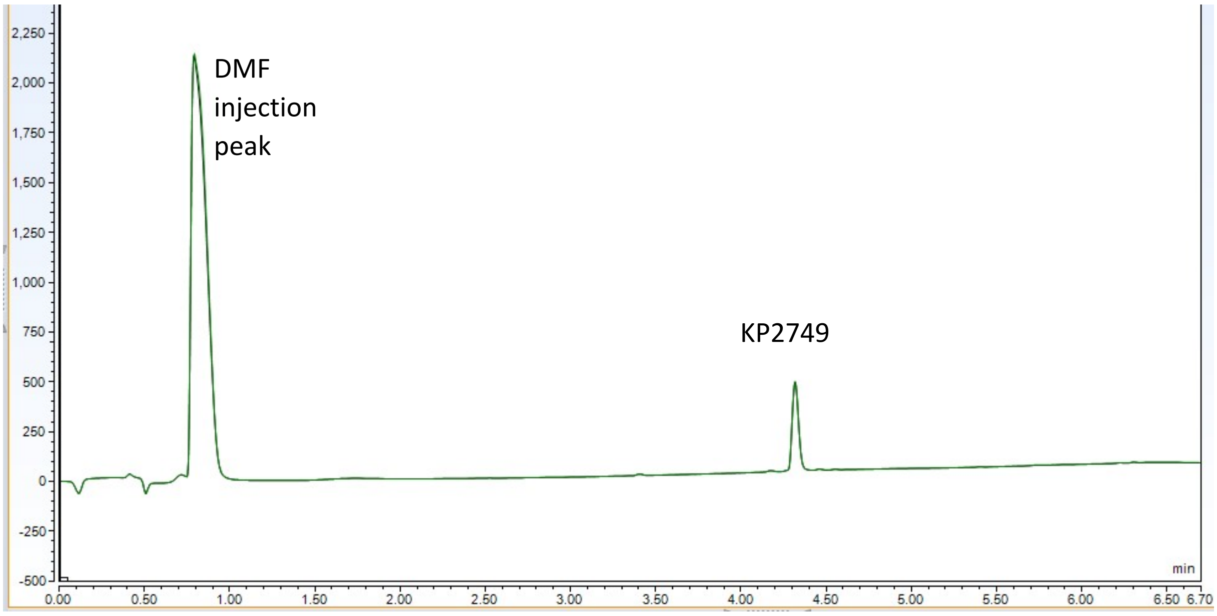

Suppl. Figure S9. UPLC chromatogram of complex KP2749.

Suppl. Table S1. Cell models and corresponding details of the cell panel used in this study.

Cell line	Tissue Type	Medium	Source
A431	Human epidermoid carcinoma	RPMI	ATCC
RU-MH	Human hypernephroma	RPMI	Established at CCR[1]
MCF-7	Human breast carcinoma	DMEM	ATCC
PC-9	Human NSCLC	RPMI	ATCC
HCC827	Human NSCLC	RPMI	ATCC
H1650	Human NSCLC	RPMI	ATCC
H1975	Human NSCLC	RPMI	ATCC
HCT116	Human colorectal carcinoma	McCoys	ATCC
HCT116/OxR	Human colorectal carcinoma	McCoys	Established at CCR[2]

ATCC, American tissue cell collection; CCR, Center for Cancer Research Vienna

References

1. Karnthaler-Benbakka, C.; Koblmüller, B.; Mathuber, M.; Holste, K.; Berger, W.; Heffeter, P.; Kowol, C. R.; Keppler, B. K. Synthesis, Characterization and in Vitro Studies of a Cathepsin B-Cleavable Prodrug of the Vegfr Inhibitor Sunitinib. *Chem. Biodivers.* **2019,** 16, e1800520.

2. Jungwirth, U.; Xanthos, D. N.; Gojo, J.; Bytzek, A. K.; Körner, W.; Heffeter, P.; Abramkin, S. A.; Jakupec, M. A.; Hartinger, C. G.; Windberger, U.; Galanski, M.; Keppler, B. K.; Berger, W. Anticancer Activity of Methyl-Substituted Oxaliplatin Analogs. *Mol. Pharmacol.* **2012,** 81, 719-28.

Suppl. Table S2. ICP-MS parameters for the measurement of platinum uptake

RF power	1550 W
Nebulizer	MicroMist
Spray chamber	Scott double-pass
Spraying chamber temp.	2°C
Monitored Isotopes	^{185}Re, ^{195}Pt, ^{196}Pt
Measurement modes	standard mode
Plasma gas	15 L min^{-1}
Nebulizer gas	1.08 L min^{-1}
Auxiliary gas	0.90 L min^{-1}
Cones	Ni
Integration time	0.1
Number of replicates	12
Number of sweeps	100
